# The Athletic Identity Measurement Scale: A Systematic Review with Meta-Analysis from 1993 to 2021

**DOI:** 10.3390/ejihpe12090097

**Published:** 2022-09-14

**Authors:** Marc Lochbaum, Sydney Cooper, Sara Limp

**Affiliations:** 1 Department of Kinesiology and Sport Management, Texas Tech University, Lubbock, TX 79409-3011, USA; 2 Institute of Educational Research, Education Academy, Vytautas Magnus University, LT-44248 Kaunas, Lithuania; 3 Track & Field, Texas Tech University Athletics, Lubbock, TX 79409-3011, USA

**Keywords:** competitive sport, quantitative review, athletics, self-perceptions, identity correlates

## Abstract

Sport psychology embraced the study of athletic identity in the 1990s. The Athletic Identity Measurement Scale (AIMS) is at the forefront of athletic identity measurement. This quantitative review examined two hypotheses: individual who are most engaged in sports identify most as athletes and thus score higher on the AIMS, and athletic identity relates to positive (e.g., intrinsic motivation) and negative (negative emotions) factors. In addition to our two hypotheses, we explored whether the AIMS subscales influenced our two hypotheses. After completing a systematic search of SPORTDiscus, APA PsycINFO, ERIC, and Psychology and Behavioral Sciences Collection APA within the EBSCOhost platform along with some hand searching, 101 articles published between 1993 and our end date of August 2021 met the inclusion criteria. The included studies investigated 20,498 athletes competing in a variety of sports from the following continents: Australia, Asia, Europe, and North America. We based all analyses on random- and mixed-effects statistics. Higher-achieving athletes, as expected, self-reported a higher degree of athletic identity. The differences between athlete groups were significant (*p* < 0.001) and meaningful (*g* values ranged from 1.55 to 1.93). The AIMS total score correlations with positive and negative factors (correlates) were small in magnitude (*r* = 0.22 and 0.17). However, the relationships differed across correlate subcategories (e.g., intrinsic motivation/commitment, *r* = 0.51, and body issues, *r* = 0.14). Minimal AIMS subscale reporting occurred across the 101 studies; thus, we could not assess their importance with certainty. In conclusion, a higher degree of athletic identity related to valued sport correlates such as intrinsic motivation/commitment and the mastery goal orientation. These correlations were small in relation to negative or less desirable factors in sport such as body disorder issues and negative emotions. We recommend future research of greater complexity and the reporting of athletes’ competitive backgrounds to understand athletic identity. In addition, researchers should report AIMS subscale data.

## 1. Introduction

William James [1] wrote on the vital role of self and identity in the human experience. Since James’ influential text, researchers, theorists, and practitioners continue to fill academic journals, textbooks, and self-help books with self-based works. Identity emerged as a standalone self-construct since the late 1960s with Erickson’s [2] text *Identity: Youth and Crisis*. In the late 1970s, Markus [3] defined specific identities as cognitive structures. Cognitive structures, referred to as self-schemas, serve to guide, with an organizational structure, incoming self-information from our lived experiences. More than one, if not more, selves exist from which a human may identify, and thus a review of this research is well beyond the scope of this review. In short, this current systematic review with a meta-analysis is specific to athletic identity, defined as “the degree to which an individual identifies with the athlete role” (p. 237) [4] as measured by the AIMS.

Brewer and his colleagues [4] popularized athletic identity in the 1990s with their publication titled *Athletic Identity: Hercules’ Muscles or Achilles Heel?* Within their publication, Brewer et al. theorized both positive (desired) and negative (not desired) factors that could associate with a strong athletic identity. The potential association with these factors was the crux of athletic identity significance, with the negative factors being of special interest. For instance, if a higher athletic identity is associated with body eating disorders, the importance of athletic identity heightens. In addition, within their publication, Brewer and colleagues presented the validation studies of what the researchers titled the AIMS. The following are the original unidimensional AIMS statements:

1. I consider myself an athlete.
2. I have many goals related to sport.
3. Most of my friends are athletes.
4. Sport is the most important part of my life.
5. I spend more time thinking about sport than anything else.
6. I need to participate in sport to feel good about myself.
7. Other people see me mainly as an athlete.
8. I feel bad about myself when I do poorly in sport.
9. Sport is the only important thing in my life.
10. I would be very depressed if I were injured and could not compete in sport.

Soon after the publication of the unidimensional AIMS, researchers [5,6] suggested a multidimensional AIMS with the following subscales: social identity (items 3 and 7), self-identity (items 1 and 2), negative affectivity (items 8 and 10), and exclusivity (items 4, 5, and 9). Next, in the early 2000s, the Brewer and Cornelius [7] refinement of the original 10 items occurred with social identity (items 1–3), exclusivity (items 4 and 5), and negative affectivity (items 8 and 10) as subscales and three statements being removed (items 6, 7, and 9).

1. I consider myself an athlete.
2. I have many goals related to sport.
3. Most of my friends are athletes.
4. Sport is the most important part of my life.
5. I spend more time thinking about sport than anything else.
6. I need to participate in sport to feel good about myself.
7. Other people see me mainly as an athlete.
8. I feel bad about myself when I do poorly in sport.
9. Sport is the only important thing in my life.
10. I would be very depressed if I were injured and could not compete in sport.

### 1.1. Athletic Identity Review

Though not specific to the AIMS, four review articles, none with meta-analyses, exist in the literature with different research questions [8,9,10,11]. Ronkanien and colleagues [8] wrote an extensive meta-study of 40 qualitative and 5 mixed-method athletic identity studies. Their work, using meta-study methodology, is furthest in content from the present systematic review with meta-analysis. In summary, the researchers sought to identify how researchers conceptualized (meta-theory) athletic identity and how the conceptualizations that are the paradigmatic assumptions and positions influenced researcher decisions regarding the methodology and finding interpretations. Ronkanien et al. provided summaries of athletic identity conceptualization (e.g., post-positivist and critical realist), the qualitative methodologies used (e.g., interviews and focus groups), and data analyses approaches (e.g., narrative analysis and thematic analysis). They concluded that, though these studies were a small portion of the meta-study, athletic identity research, both qualitative and quantitative, must be explicit concerning philosophical underpinnings and grounded more in psychology-based identity theory.

Renton et al. [10] conducted a scoping review concerned with athletic identity and sport-related injury outcome measures, such as physical functioning, pain, and psychosocial outcomes. As with the current review, Renton et al. restricted their review to studies with the AIMS. The researchers reviewed 22 studies with 1852 athletes. With extensive demographic details as well as injury-related outcomes, Renton et al. concluded that the AIMS related to all the outcome categories: behavioral, psychosocial, and injury-specific. Given the scoping review methodology, the researchers did not calculate the meaningfulness of the relationships. Renton et al. noted the 22 studies lacked theoretical injury models and diverse samples.

Whereas Renton et al. examined athletic identity in the injury environment, Steele and colleagues [9] reviewed student athletes’ athletic identities in the academic environment. With an initial sample of 42 studies, the researchers presented main findings for their included studies, focused on 16 studies with both athletic and academic identities directly or indirectly measured. Steele et al. concluded the literature themes to date include identity development, career development, motivation, role conflict, and student-athlete stereotypes. The researchers concluded a greater need for mixed-method studies and longitudinal studies to best understand the interplay of both identities on the student athletes’ performances and wellbeing.

In the last of the reviews, Edison and colleagues [11] sought to present a systematic review of the epidemiological characteristics of athletes’, aged 22 or younger, athletic identity. Athletic identity measurement was not specific to just the AIMS. The authors reviewed the 10 included studies on the following: demographics, participation in sports and physical activity, injury, and mental health. Though the authors suggested athletic identity differs by race/ethnicity and career state, the number of studies limited strong conclusions. Of most relevance to the current review, Edison et al. concluded higher athletic identity projected the athletes against burnout, a negative factor, in participating athletes and depression in injured athletes.

### 1.2. Research Aims

The past reviews provided insights into the athletic identity literature. However, no review to date examined Brewer and colleagues’ original propositions or hypotheses. Hence, we sought, with meta-analytic methods and analyses, to address Brewer and colleagues’ two main AIMS hypotheses: first, individual who are most engaged in sports (e.g., a career or a sizeable portion of their daily lives) will identity most as an athlete and thus score higher on the AIMS, and second, athletic identity might relate to positive (Hercules’ muscles) and negative (Achilles heel) factors. Last, we sought to examine whether the AIMS subscales, not designed first by Brewer and his colleagues, influenced one or both of our main results. We did not put forward hypotheses as to the potential influence of subscales on our main hypotheses.

## 2. Materials and Methods

This systematic review with meta-analysis followed the PRISMA statement [12] (see Supplementary Materials Table S1 for the PRISMA checklist corresponding to our review).

### 2.1. Eligibility Criteria

Eligible articles met the following criteria for inclusion for the AIMS and AIMS subscale analyses: (a) athletic participants competing during the time of questionnaire completion; (b) peer-reviewed journals containing mean AIMS or subscale data scored on a 1 to 7 Likert system; and (c) a valid Brewer AIMS questionnaire. For the correlation analyses, eligibility criteria for inclusion were (a) athletic participants competing during the time of questionnaire completion; (b) peer-reviewed journals containing correlation data between the AIMS total score or subscales and a correlate; and (c) a valid Brewer AIMS questionnaire. Our specific exclusion criteria for the participants included injured participants or retrospective data collections (e.g., retired athletes thinking back to when they were competing). In addition, we excluded articles associated with injury rehabilitation or post-surgery data. Last, we did not impose a language of publication restriction with the note that we only searched with English language keywords.

### 2.2. Information Sources and Search Strategy

We conducted the search in EBSCO with the following individual databases: SPORTDiscus, APA PsycINFO, ERIC, and Psychology and Behavioral Sciences Collection. The first search, completed by SL and ML, concluded in May 2019. SC and ML examined the first search and expanded the search to August 2021. In both searches, we used the following search terms: athletic identity measurement scale and sport*. In EBSCO, we used the advanced search option that provides separate boxes for search terms, such as box 1 (athletic identity measurement scale), box 2 (sport*), and box 3 (N/A). At each stage, we restricted EBSCO to a one-year period (e.g., 1993). After exhausting a given year, we moved to the next year (e.g., 1994). The complete record of our search and records is available from ML. The following is an example of the 1995 search strategy:

1. Delimited search to 1995;
2. Box 1: athletic identity measurement scale;
3. Box 2: sport*.

Of three results, we selected two.

### 2.3. Article and Data Selection Process and Data Items

**Figure 1:**
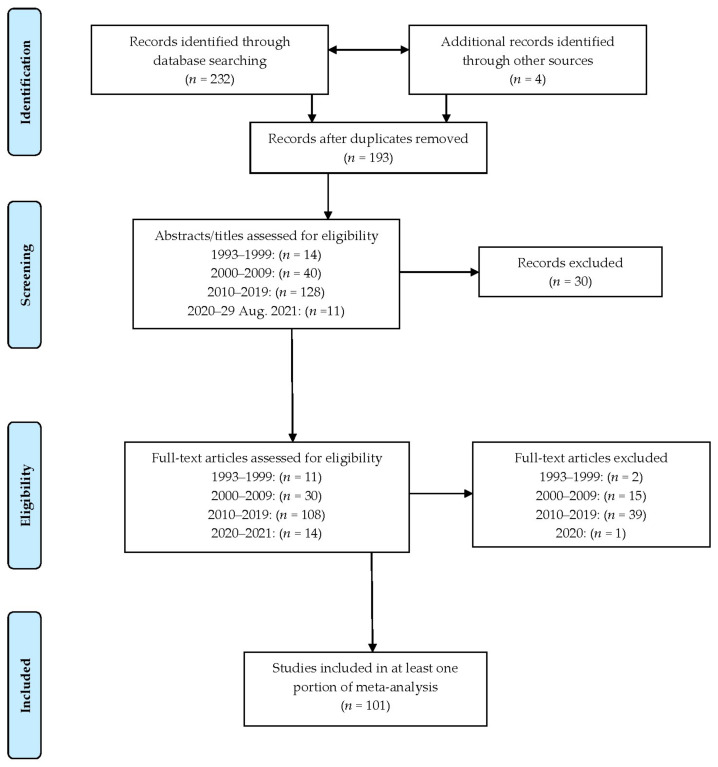
PRISMA flow chart.

Our search began before the PRISMA 2020 (http://prisma-statement.org/prismastatement/flowdiagram.aspx) (last accessed for website accuracy on 27 April 2022) updated search figures; hence, we used pre-2020 PRISMA search flow chart (Figure 1). In groups, SL and ML completed the study selection process, and then SC and ML reviewed the past search and restarted the search. Through the process, we settled disagreements by consensus. SL and ML developed the data extraction template. Again, in the same pairs (SL/ML and SC/ML), each followed the same extraction process. Given the time covered (i.e., data storage was unlikely and there was the potential for deceased authors), we did not seek data from authors.

We extracted the following information: participant athlete-level information (eventually coded as elite, advanced, intermediate, youth, and mix, see Table 1 for the coding system), country, age (mean or range), gender (percent females in sample), AIMS version (1993 or 2001), number of items (7 or 10 were the dominant versions), correlate questionnaire title, and data available (mean level and correlation). We also extracted the sport name or names for each sample. For the mean level analyses, we extracted the mean, standard deviation, and sample size. For the correlate portion of our review, we extracted all correlation values and the sample size. We wrote the correlate questionnaire name in the extraction file to aid in identifying and grouping positive and negative factors consistent with Brewer and colleagues’ [4] review of such factors (e.g., self-worth, emotions, and participation motivations).

### 2.4. Risk of Bias Assessments

SL and SC coded the studies on the following individual study risks of bias: data sampling methods (i.e., convenience or a form of random sampling), data collection methods (i.e., in-person or not), and the reported AIMS study-level reliability values (i.e., yes or no). ML discussed all coding in pairs (SL/ML and SC/ML). We planned to examine whether either risk moderated our mean level and correlate results. For risk of bias across studies, we examined publication bias with the following: the classic fail-safe *n*, Orwin’s fail-safe *n*, the funnel plot, and the ‘trim and fill’ results. The classic fail-safe *n* statistic represents the number of null samples required to change a significant value into a non-significant value [15]. Orwin’s fail-safe *n* is not identical to the classic fail-safe *n* because Orwin’s fail-safe *n* is the number of potential missed studies that, when added to the actual data, would move the new correlation past a chosen threshold [16]. We chose *r* = 0.00 as our missed study value and *r* = 0.10 as our threshold, as this value is the lower end of a correlation with low meaningfulness. Hence, the greater the value for both fail-safe *n* calculations, the greater the confidence that the result is safe from publication bias. We specified the one-tail test when we conducted the classic fail-safe *n* analysis. To see whether the entered studies dispersed equally on either side of the overall effect, we examined funnel plots [17]. Full plot symmetry represents that the retrieved studies captured the essence of all studies. Last, we examined Duval and Tweedie’s [18] trim and fill analysis. If required, data points filled to the right increase the effect size value, whereas those filled to the left lower the effect size value.

### 2.5. Effect Size Measures, Synthesis Methods, and Certainty Assessment

Given the straightforwardness of our analyses, we entered the mean AIMS values and correlation coefficients. We interpreted the correlation values as follows: 0.10–0.29 as small, 0.30–0.49 as medium, and 0.50 or greater as large [19]. To assess the meaningfulness of mean level differences, we calculated Hedges’ g, and followed standard guidelines, with 0.20 as small, 0.50 as medium, 0.80 as large, and 1.30 as very large. We assumed heterogeneity, as heterogeneity exists in sport psychology meta-analyses [20]. Thus, we planned both random- and mixed-effects analyses. We reported the number of cases, sample size, *r*, 95% confidence intervals, heterogeneity, and publication bias statistics for our analyses. We reported the *I*^2^ statistic, the ratio of excess dispersion to total dispersion, as our heterogeneity measure with the following interpretation: <25 (low), at least 50 (medium), and >75 (high) [21]. For our moderator tests, we used a mixed-effects analysis. For these analyses, we reported the number of cases, sample size, *r*, 95% confidence intervals, and the Q total between (Q_TB_) with an associated *p*-value. We set the statistical significance at the traditional *p* < 0.05. The Q_TB_ indicates the level of difference between different moderator levels. We conducted our meta-analyses using the Comprehensive Meta-Analysis (CMA) version 3 software (version 3.3.070, Biostat, Inc., Englewood, NJ, USA) and ran our descriptive analyses with Intellectus Statistics (https://www.intellectusstatistics.com/). Last, we examined our results (e.g., confidence intervals, sample sizes, and differences between groups and correlate categories) with the aim of assessing certainty.

## 3. Results

### 3.1. Study Selection and Characteristics

From the PRISMA-guided search (refer to Figure 1), 101 studies met all inclusion criteria. Table 2 includes the 101 studies meeting all inclusion criteria. The studies spanned from 1993 to 2021, including 20,498 participants with data coming from the following continents: Australia—Australia; Asia—China, Israel, Iran, Japan, and Taiwan; Europe—Finland, Germany, Ireland, Italy, France, Greece, Poland, Slovenia, Spain, Sweden, and The Netherlands; and North America—Canada and the United States of America. We coded samples without enough information to code the exact countries as Mix. The studies varied in the percentage of females involved in the study, including none (*n* = 25), more than none to 50% (*n* = 40), more than 50 to below 100% (*n* = 22), all (*n* = 7), and not reported (*n* = 6). Of the sample participants ages, more than half were aged from 18 to 30 (*n* = 54). The rest of the samples were under 18 (*n* = 26), above 30 (*n* = 13), or not reported (*n* = 8). As coded, the studies spanned participant competition levels, including advanced (*n* = 39), elite (*n* = 22), mixed (*n* = 16), intermediate (*n* = 14), youth (*n* = 5), and recreational (*n* = 5). Concerning the AIMS questionnaire version, they were about even, with 50 using the 2001 version and 51 using the 1993 version. Last, concerning the data used in our meta-analysis, 8 studies provided only correlations, 57 provided mean data, and 37 provided mean and correlation data.

### 3.2. Risk of Bias within Studies

Table 3 provides information on the risk of bias within studies. The major concern is the method of sampling, as 99 of the 101 studies used a convenient sample. Thus, with just this information, the studies are of low quality, consistent with cross-sectional data in sport psychology [20]. We examined whether the data collection method and AIMS study-level reliability reporting moderated our results. No significant differences resulted in the mixed-effects analyses (i.e., the data collection method for all AIMS score, subscales, and AIMS scores by athlete subgroups and the AIMS study-level reliability reported again for AIMS total score, subscales, and AIMS scores by athlete subgroups).

Though not reaching traditional significance (i.e., *p* < 0.05), a trend emerged with the correlations and the data collection bias risk, suggesting an impact of the data collection method (i.e., more favorable perceptions in person). The AIMS total score and positive factors correlation for in-person data collection, *r* = 0.27, 95% CI [0.12, 0.39], was greater than when not in-person, *r* = 0.11, 95% CI [−0.07, 0.30]. For the negative factor correlates, the pattern reversed, in that the in-person data collection, *r* = 0.11, 95% CI [−0.05, 0.19] was smaller than when not in-person, *r* = 0.22, 95% CI [0.12, 0.30]. All correlate studies reported study-level reliability statistics; hence, analyses were not possible.

**Table 3:** Risk of individual study bias questions for all included studies.

Study Information		Risk of Study Bias Questions		
Author(s) [Ref#]	Year	Sampling ^1^	Collection ^2^	Reliability ^3^
Ohji et al. [22]	2021	Yes	In-person	No
Geary et al. [23]	2021	Yes	In-person	No
Hagiwara [24]	2020	Yes	In-person	Yes
Graham and Burns [25]	2020	Yes	Not in-person	Yes
Costa et al. [26]	2020	Yes	Not in-person	Yes
Koper et al. [27]	2020	Yes	In-person	Yes
Moazami-Goodarzi et al. [28]	2020	Yes	Not in-person	Yes
Graupensperger et al. [29]	2020	Yes	Not in-person	Yes
Ioannis [30]	2020	No (purposeful)	In-person	Yes
Rongen et al. [31]	2020	Yes	Not in-person	No
Walsh et al. [32]	2020	Yes	Not in-person	Yes
Roethlisberger et al. [33]	2020	Yes	In-person	Yes
Samuel et al. [34]	2020	Yes	Not in-person	Yes
Hadiyan and Cosh [35]	2019	Yes	In-person	Yes
Kola-Palmer et al. [36]	2019	Yes	Not in-person	Yes
Pummell and Lavallee [37]	2019	Yes	In-person	Yes
Piatt et al. [38]	2018	Yes	Not in-person	Yes
Voelker et al. [39]	2018	Yes	In-person	Yes
Bell et al. [40]	2018	Yes	Not in-person	Yes
Franck et al. [41]	2018	Yes	In-person	Yes
Chang et al. [42]	2018	Yes	In-person	Yes
Prioios [43]	2017	Yes	In-person	Yes
Giannone et al. [44]	2017	Yes	Not in-person	Yes
O’Rourke et al. [45]	2017	Yes	In-person	Yes
Turton et al. [46]	2017	Yes	Not in-person	Yes
Geukes et al. [47]	2017	Yes	In-person	Yes
Fuller [48]	2017	Yes	Not in-person	Yes
Van Lone et al. [49]	2017	Yes	Unable to determine	Yes
Peiró-Velert et al. [50]	2016	Yes	In-person	Yes
Nagata and Long [51]	2016	Yes	In-person	Yes
Haralabos et al. [52]	2016	Yes	Not in-person	Yes
Samuel et al. [53]	2016	Yes	In-person	Yes
Franck et al. [54]	2016	Yes	In-person	Yes
Huang et al. [55]	2016	Yes	In-person	Yes
Waldron [56]	2015	Yes	Not in-person	Yes
Samuel et al. [57]	2015	Yes	In-person	Yes
Poux and Fry [58]	2015	Yes	Not in-person	Yes
Houle and Kluck [59]	2015	Yes	Not in-person	Yes
Schutte and McNeil [60]	2015	No (used panel system)	Not in-person	Yes
de Subijana et al. [61]	2015	Yes	Unable to determine	Yes
Kroshus et al. [62]	2015	Yes	Not in-person	Yes
Stambulova et al. [63]	2015	Yes	In-person	Yes
Hagiwara and Isogai [64]	2014	Yes	Unable to determine	No
Nagata [65]	2014	Yes	Not in-person	Yes
Harris and Watson [66]	2014	Yes	In-person	No
Price et al. [67]	2014	Yes	Not in-person	Yes
Voelker et al. [68]	2014	Yes	In-person	Yes
Madrigal and Gill [69]	2014	Yes	In-person	Yes
Mitchell et al. [70]	2014	Yes	In-person	No
Bimper [71]	2014	Yes	In-person	Yes
Poczwardowski et al. [72]	2014	Yes	In-person	Yes
Petrie et al. [73]	2014	Yes	In-person	Yes
Martin et al. [74]	2014	Yes	Not in-person	Yes
Weinberg et al. 2013 [75]	2013	Yes	Not in-person	Yes
Proios [76]	2013	Yes	In-person	Yes
Tyrance et al. [77]	2013	Yes	Not in-person	Yes
Martin and Horn [78]	2013	Yes	In-person	Yes
McKay et al. [79]	2013	Yes	In-person	No
Tasiemski et al. [80]	2013	Yes	In-person	Yes
Verkooijen et al. [81]	2012	Yes	Not in-person	No
Wiśniowska et al. [82]	2012	Yes	Unable to determine	Yes
Tasiemski et al. [83]	2012	Yes	Unable to determine	Yes
Steinfeldt and Steinfeldt [84]	2012	Yes	Not in-person	Yes
Harrison et al. [85]	2011	Yes	Unable to determine	Yes
Kissinger et al. [86]	2011	Yes	Not in-person	Yes
Samuel and Tenenbaum [87]	2011	Yes	In-person	Yes
Sturm et al. [88]	2011	Yes	Not in-person	Yes
Steinfeldt et al. [89]	2011	Yes	In-person	Yes
Gapin and Petruzello [90]	2011	Yes	In-person	Yes
Visek et al. [91]	2010	Yes	In-person	Yes
Chen et al. [92]	2010	Yes	In-person	No
Lau et al. [93]	2010	Yes	Not in-person	Yes
Mateos et al. [94]	2010	Yes	In-person	Yes
Packar [95]	2010	Yes	Not in-person	Yes
Caudroit et al. [96]	2010	Yes	In-person	Yes
Steinfeldt and Steinfeldt [97]	2010	Yes	In-person	Yes
Steinfeldt et al. [98]	2010	Yes	In-person	Yes
Maxwell and Visek [99]	2009	Yes	Not in-person	Yes
Groff et al. [100]	2009	Yes	Not in-person	Yes
Kokaridas et al. [101]	2009	Yes	In-person	Yes
Steinfeldt et al. [102]	2009	Yes	Not in-person	Yes
Mateos et al. [103]	2008	Yes	In-person	Yes
Mignano et al. [104]	2006	Yes	In-person	Yes
Phoenix et al. [105]	2005	Yes	In-person	Yes
Albion and Fogarty [106]	2005	Yes	Not in-person	Yes
Lau et al. [107]	2004	Yes	In-person	Yes
Tasiemskie et al. [108]	2004	Yes	Not in-person	No
Grove et al. [109]	2004	Yes	Both	Yes
Schmid and Seiler [110]	2003	Yes	Unable to determine	Yes
Kornspan and Etzel [111]	2001	Yes	Not in-person	Yes
Horton and Mack [112]	2000	Yes	Not in-person	Yes
Martin [113]	1999	Yes	Not in-person	Yes
Lantz and Shroeder [114]	1999	Yes	In-person	Yes
Hale et al. [115]	1999	Yes	Not in-person	No
Smith et al. [116]	1998	Yes	Not in-person	Yes
Wiechman and Williams [117]	1997	Yes	Not in-person	Yes
Murphy et al. [118]	1996	Yes	In-person	Yes
Martin et al. [6]	1995	Yes	Not in-person	Yes
Cornelius [119]	1995	Yes	Not in-person	Yes
Brewer et al. study 1 [4]	1993	Yes	In-person	Yes
Brewer et al. study 3 [4]	1993	Yes	In-person	Yes

^1^ Was the sampling convenient? ^2^ Were data collected in-person or online or a combination? ^3^ Were AIMS questionnaire reliability statistics reported at the study level?

### 3.3. AIMS Total and Subscale Results

**Figure 2:**
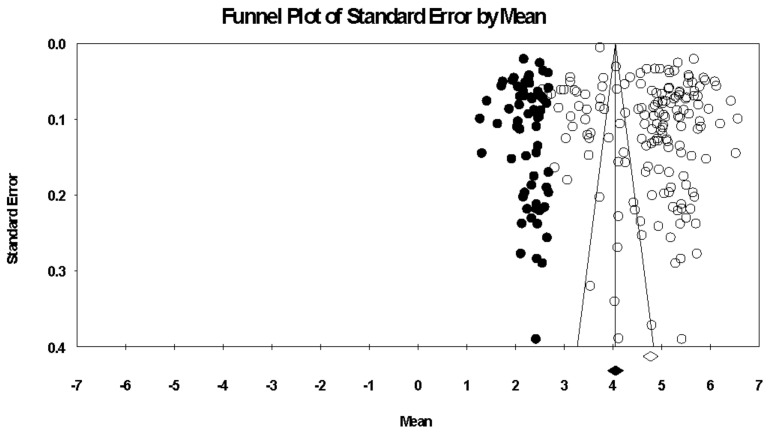
Random-effects funnel plot of standard error of the mean for the AIMS total scores. Clear circles are the observed data; filled-in circles are the imputed data.

**Figure 3:**
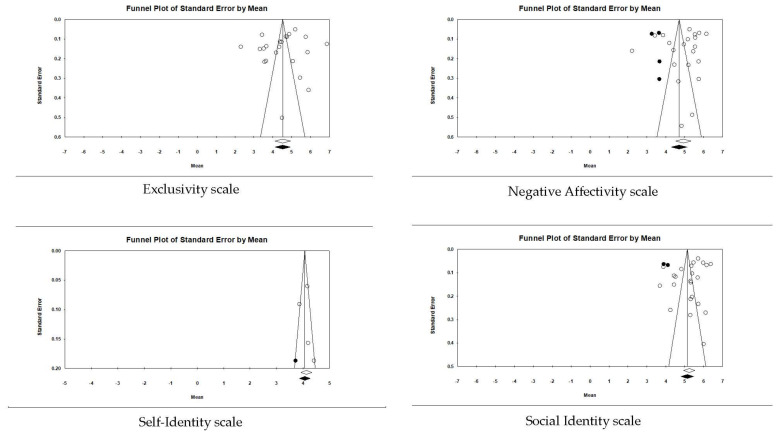
Random-effects funnel plot of standard error of the mean for the four AIMS subscales. Clear circles are the observed data; filled-in circles are the imputed data.

**Figure 4:**
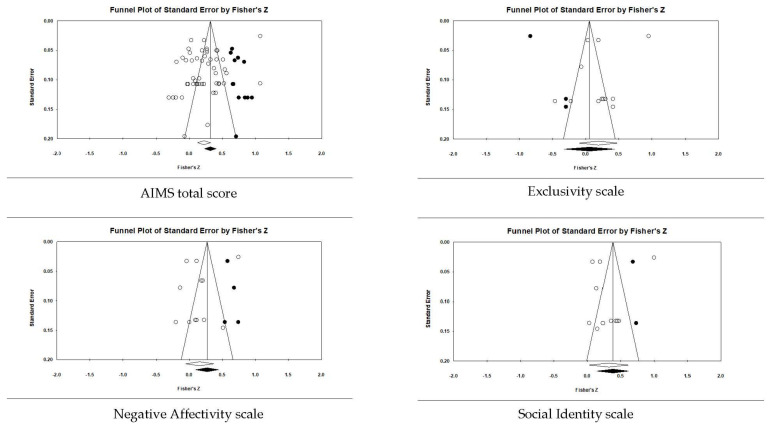
Random-effects funnel plot of standard error of the mean for positive (desired) correlates with AIMS total score and subscales. Note that there were not enough data to run the trim and fill analysis for the self-identity scale. Clear circles are the observed data; filled-in circles are the imputed data.

**Figure 5:**
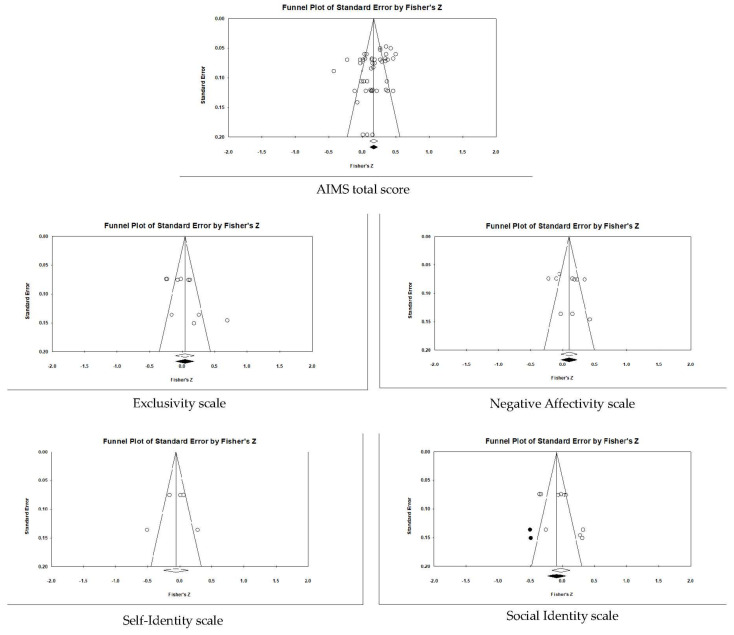
Random-effects funnel plot of standard error of the mean for negative (undesired) correlates with AIMS total scores and subscales. Clear circles are the observed data; filled-in circles are the imputed data.

**Figure 6:**
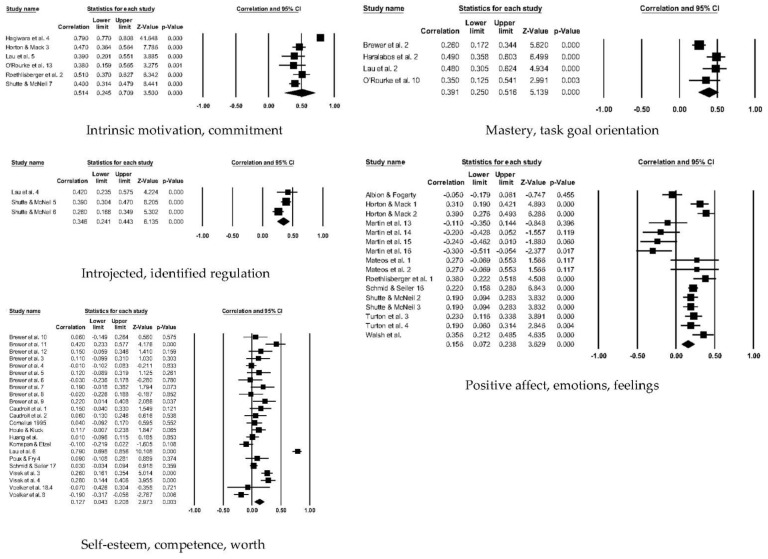
Random-effects individual correlates for AIMS total score with the positive factors. References listed as appear per correlate forest plot. Studies repeated within a correlate listed only once. *Intrinsic motivation, commitment* [24,33,45,60,107,112]. *Mastery, task orientation* [4,45,52,107]. *Introjected, identified regulation* [60,107]. *Positive affect, emotions, feelings* [6,32,33,46,60,94,103,106,110,112]. *Self-esteem, competence, worth* [4,39,55,58,59,68,91,96,107,110,111,119].

Before examining our first hypothesis and the potential importance of the AIMS subscales, we examined the overall pattern of the scale values for all participants. Supplementary Materials Table S2 contains data for each study, and Table 4 contains the data for all samples. The AIMS total scores and subscale scores ranged between 4.13 and 5.24. When examining publication bias, there was little bias in the data (see Figure 2, Figure 3, Figure 4, Figure 5 and Figure 6). The scales requiring trim and fill showed a slight downward trend, suggesting publication bias towards higher scores. The importance of athlete level (see Table 5) suggests the publication bias result is due to the types of participants studied. It is possible that more elite athlete data were published, and lower-level athlete AIMS data were rejected. From our effect size statistics, we justified our use of the random-effects model for the remaining analyses, as all scales, excluding the self-identity subscale, had high heterogeneity (*I*^2^) values.

Next, we examined whether mean differences existed between or among the athletes’ achievement levels. Consistent with Brewer and colleagues [4], we hypothesized that athletes competing in elite and advanced sports would endorse a higher AIMS total mean value than all other athlete groups and the differences would become more noticeable compared to recreational and youth athletes and perhaps the intermediate category of athletes. We only examined the AIMS total score, as the subscale samples were few (see Supplemental Materials Table S2). For this analysis (see Table 5 for statistics), we excluded the ‘mix’ participant level. We ran a group mixed-effects analysis for the AIMS total and calculated Hedge’s *g* to determine the meaningfulness of the differences between athlete categories. The group mixed-effects analysis was significant (*p* < 0.001); the recreational and youth 95% CI upper limits did not overlap with the elite, advanced, and intermediate 95% CI lower limits. Hedge’s *g* for the elite, advanced, and intermediate athlete means were all very large (*g* values ranged from 1.55 to 1.93) compared with the recreational and youth athletes.

### 3.4. Correlate Results

We extracted over 500 individual correlations. Of those, 170 (see Supplemental Materials Table S3 for all included individual study data in table form) fit our interpretation of Brewer and colleagues’ [4] positive and negative factors. We excluded environmental correlates such as achievement goal climate, personality constructs, and others (e.g., grade point average) that surround the athletic environment but did not fit within Brewer and his colleagues’ factors. All correlations, by study with correlate questionnaire names, are available from ML.

Table 6 contains the effect sizes and publication bias statistics for the correlate analyses. Figure 4 (positive factors) and Figure 5 (negative factors) depict the publication bias trim and fill results. For the positive factor correlates, meaningfulness ranged from small (AIMS total score, negative affectivity, and exclusivity) to medium (social identity, and self-identity). When considering publication bias, the AIMS total score changed to a medium meaningfulness interpretation, whereas exclusivity changed from small to negligible meaningfulness. The classic fail-safe *n* and Orwin’s *n* statistic suggested the mean correlations, where applicable, would require a substantial number of studies to change. In contrast to the positive factor correlations, only the AIMS total score and the negative factor values were small in meaningfulness, with a significant Z value. Though smaller when compared to the positive factor bias statistics, the classic fail-safe *n* and Orwin’s *n* statistic indicated the AIMS total score and negative factor correlations are free of publication bias based on the number of studies required for change.

**Figure 7:**
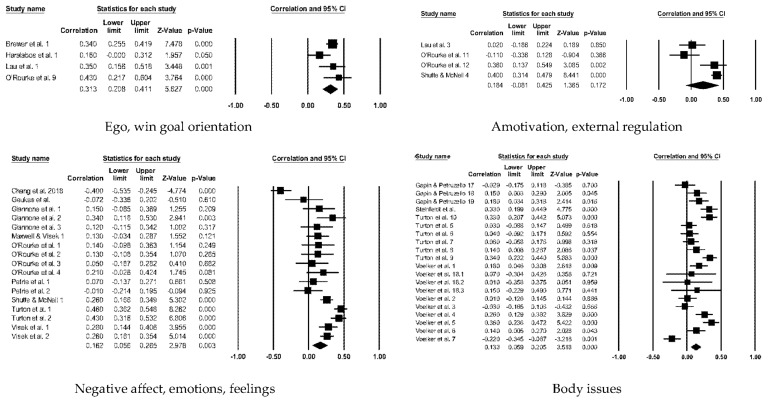
Random-effects individual correlates for AIMS total score with the negative factors. References listed as appear per correlate forest plot. Studies repeated within a correlate listed only once. *Ego, win goal orientation* [4,45,52,107]. *Amotivation, external regulation* [45,60,107]. *Negative affect, emotions, feelings* [42,44,45,46,47,60,73,91,99]. *Body issues* [39,46,68,89,90].

To better understand our overall positive (Figure 6) and negative factor (Figure 7) correlations, we examined the correlate subcategories (see Table 7) with the AIMS total score. All the mean random-effects correlations were significantly different from zero, except the amotivation and external regulation category. The intrinsic and commitment correlation was large, the positive factor subcategories mastery/task goal orientation and introjected/identified regulations as well as the negative factor category ego/win goal orientation were medium correlations, and the rest of the subcategories had low meaningfulness.

## 4. Discussion

Brewer and colleagues [4] accelerated the athletic identity literature in the USA and around many parts of the world by publishing their 10-item AIMS. This systematic review with meta-analysis assessed their original thoughts as to who would endorse such an identity and how athletic identity might relate to positive (Hercules’ muscles) and negative (Achilles heel) factors. To date, no such review exits in the literature, as the previous four [8,9,10,11] reviews addressed different questions. In addition to testing Brewer et al.’s basic premises, we sought to examine whether the AIMS subscales affected our main findings. However, with limited subscale reporting, our ability to analyze the subscales, with the exception of the possibility that the social identity subscale is of value, was limited. We provide limited thoughts in our discussion, with a note that during the revision process the first author (M.L.) received from (email correspondence, 23 August 2022) Britton Brewer (B.B.) his in-press third-generation AIMS measure. In our conclusions section, we discuss this new measure. Before doing so, we address our research hypotheses regarding the certainty of evidence and provide some future research ideas.

### 4.1. AIMS Score Differences

Our first research question addressed the following hypothesis: individuals who are most engaged in sports will identity most as athletes and thus score higher on the AIMS. We coded for the following standard of performance categories: elite, advanced, intermediate, recreational, and youth. Our results confirmed participants at the higher achievement standards, and thus assumed to be most invested in athletics, identified most with the AIMS, as the mean AIMS values ordered from the highest level (elite) down to the lowest level (youth). Very large effect size differences among elite, advanced, and intermediate and recreational and youth. Thus, we conclude with high certainty that individuals who are most engaged in sports identify most as athletes. Thus, the AIMS discriminates among athletes as intended.

Commitments in terms of time, energy, and resources are great to achieve lofty standards in all life domains. The typical assumption is that reaching the top in athletics requires more commitment than it does to be in lower levels of athletics (e.g., regional competitions). An alternate hypothesis or interpretation is that athletes who we, as sport consumers, view as the most successful (e.g., high regard for the Olympic Games over that of a local city competition) feed into our perception and the media coverage defining success and thus they identify more as athletes. Certainly, the age of entry into sports and maturity are considerations. Youths less than 19 years of age are not eligible for the National Basketball Association (NBA) and, of course, a 10-year-old is not physically mature enough to play in the NBA. The athlete may be committed and spend lots of energy in his or her sport but is simply not eligible for the higher standard.

The included literature lacked information to complete Swann et al.’s [14] taxonomy, which considers factors that would help us to understand athletic identity formation. Those factors are an athlete’s highest standard of performance, success at the athlete’s highest level, experience at the athlete’s highest level, competitiveness of sport in an athlete’s country, and the global competitiveness of a sport. Researchers coding for all aspects of Swann and colleagues’ taxonomy will enrich future research with the AIMS. A great deal of information exists in the taxonomy that could enhance our understanding of athletic identity formation. Though it is most probable that higher levels of commitment are required for top-level athletic performances, and thus identifying as an athlete is a logical result, information such as years competing at each level or the importance of the sport in an athlete’s country seem valuable. Researchers considering longitudinal research along with the taxonomy information seems to be the best practice.

### 4.2. AIMS Correlates

Our second purpose concerned testing how athletic identity might relate to positive (Hercules’ muscles) and negative (Achilles heel) factors. Our overall positive and negative factor correlations appeared, at the outset, to be small in meaningfulness; thus, in essence, higher levels of athletic identity neither hurt nor helped. Even being small, the correlations were reliably different from zero. Hercules’ muscle and Achilles heel might balance one another out. However, with bias considered, Hercules’ muscle and the AIMS total score crossed into the medium level of meaningfulness. Publication bias statistics suggested a bias-free Achilles heel relationship. Thus, certainty is high that the AIMS relates to both positive (Hercules’ muscles) and negative (Achilles heel) factors.

The subcategory analyses provided evidence that a higher athletic identity related to Hercules’ muscles much more than Achilles heels. The evidence indicated the valued motivation constructs, all of which are themselves correlated [120], correlated higher than the average positive factor correlated with the AIMS total score. Higher levels of athletic identity and intrinsic motivation and commitment are a basic premise [4] that are discussed much in this review. Success takes time, and time requires commitment to and enjoyment of the required tasks. Both goal orientations related to a higher athletic identity. Though there is no compelling evidence that more elite athletes are higher in either achievement goal orientation than less elite athletes, it is the view taken across the literature [121]. It is more important that, while the mastery goal orientation is related to many desired correlates in sport, the ego goal orientation is not related to any meaningful degree to less desired correlates in sport [120].

The other correlate subcategories we extracted from the included studies were small in meaningfulness. Dismissing these positive and negative factor correlates is not our intent. Body issues (e.g., disordered eating behaviors) might relate to a higher athletic identity in aesthetic sports. A line of research into a specific type of sport to our knowledge does not exist. A mix of sports with a variety of levels of achievement could be the reason for our findings for body issues, positive and negative emotions, and even our self-variable category, whereas intrinsic motivation and commitment are constructs that are not dependent upon the type of sport. More focused research is required in a few areas. Body issues as well as mood seem to be important [122].

It is important to consider the risk of individual study bias, as the results suggested under-reporting of the negative factors when completing the questionnaires in-person and over-reporting the positive factors. The potential for a stronger relationship with the AIMS and negative factors and a weaker relationship with positive factors could depend on the setting. Whether this under- or over-reporting occurred, there were not enough samples with in-person and not in-person data collection methodology to examine with our subcategories. Future research could seek to understand the AIMS relationship with the positive and negative factors with the appropriate research designs. As mentioned, with the AIMS scores and the athlete categories, longitudinal research is an essential next step. Perhaps disordered body issues correlate more with the AIMS during maturation transitions and with critical steps in moving from lower-level sport settings to more elite settings.

### 4.3. AIMS Literature and Study Limitations

The AIMS literature limitations stem from a lack of random participant selection, a lack of reporting the AIMS subscales, a lack of standard athlete specifics, and all issues concerning a clear theoretical framework, as mentioned in past reviews. Hence, those issues require no more elaboration, except that of the AIMS subscales. Given the lack of reporting of subscale values and correlations with measured factors, their values are unknown. Our examination was pure speculation in that we formed no hypotheses of how they would influence our main analyses. Brewer and his colleagues’ [123] new measure compels us to believe the past subscales are just that: in the past. The new measure contains the following: a 4-item unidimensional athletic identity scale and two new subscales with two factors each, titled athletic identity properties (prominence and self-worth contingency) and athletic identity processes (self-presentation and social reinforcement).

Concerning our study, the PRISMA statement provides a concrete pathway for performing a systematic review with meta-analysis. Even so, limitations exist in our writing and following the PRISMA statement. We coded 101 studies. We eliminated a handful of studies for our AIMS score differences analyses because the authors used 1 to 5 (*n* = 5) or 1 to 6 (*n* = 1) Likert scales, which were not analyzable on their own or with the 1 to 7 Likert-scored studies. Our attempt to include non-English-language studies is a potential source of missed studies. In our search, we wrote in English. The EBSCO search thus scanned for English words in the article titles, abstracts, and keywords. We did not search in foreign languages. Our next few study limitations seem more important than missing studies, with 101 included. Being able to report participant expertise level is a study limitation. We attempted, at the outset, to utilize only Swann and his colleagues’ [14] taxonomy for classifying athletic samples. The research literature before their 2015 publication, of course, did not use their taxonomy. We did not find the taxonomy used in our included studies since 2015. Swann and colleagues’ taxonomy requires a good deal of information. Our attempts to use the taxonomy would require assumptions. Hence, we used information from the taxonomy [14] and a past goal-setting meta-analysis [13] to best categorize the athletes. We suggest the use of this taxonomy in all competitive sport research. Last, though extensive risk of study-bias rating systems exists, our three rated risks might be limitations. However, the AIMS literature is cross-sectional. Thus, without any random sampling procedures as the minimum, any rating system is descriptive and with little potential to determine the impact on our two main research questions.

## 5. Conclusions

The athletic identity literature is extensive, with several past reviews summarizing different research questions and samples. Our meta-analyzed results demonstrated greater involvement in athletics and thus identification related strongest to Hercules’ muscles (positive factors) than Achilles heels (negative factors). Our work is unique and furthers the athletic identity literature specific to the AIMS. When thinking of practical recommendations, the AIMS total score is a useful assessment tool for continued work in athletic identity. The need for subscale scores seems limited. With the new third-generation measure, the new subscales will be at the forefront of AIMS research. As mentioned, longitudinal research with positive and negative factors with the AIMS will further the literature scope. Identifying potential vital moments (e.g., transitions from youth to intermediate/advanced athletics) is non-existent in the literature. Even with limitations and needs for future research, akin to Hercules’ twelve labors, athletes should seek athletic adventures in earnest, as the upsides outweigh the downsides of earnest participation.

## Figures and Tables

**Table 1:** Athlete-level categories.

Category	Category Specifics
Elite	Olympics, world championships, international competition, professional, and samples >18 years of age
Advanced	College athletes in all countries, youth/adolescents in talent programs (e.g., sport schools), beyond high school or local city club team, and national-level competition
Intermediate	14–18 years of age, USA high school, club, not identified as elite or in college, etc., but in extensive training and regional-level competition
Recreational	College intramural and city teams
Youth	Below high school, not identified as elite in some way, and sample mean age <14
Mix	Unable to determine one category for sample data

Note: Categories based on Kyllo and Landers [13] and Swann et al. [14].

**Table 2:** Participant characteristics and AIMS information for studies meeting inclusion criteria.

Study Information		Participant Characteristics				AIMS Information		
Author(s) [Ref#]	Year	Level	% Female	Country	Age	Version	Items	Data
Ohji et al. [22]	2021	M	52.30	JP	20.00	2001	7	M
Geary et al. [23]	2021	E	0.00	IE	NR	1993	10	M
Hagiwara [24]	2020	I	0.00	JP	19.18	2001	7	M, r
Graham and Burns [25]	2020	M	55.00	US	20.00	2001	7	M
Costa et al. [26]	2020	M	52.80	IT	27.41	2001	7	M
Koper et al. [27]	2020	E	22.00	Mix	29.80	2001	7	M
Moazami-Goodarzi et al. [28]	2020	A	51.00	FI	16.00	2001	10	M, r
Graupensperger et al. [29]	2020	A	63.00	US	19.84	2001	10	M
Ioannis [30]	2020	M	23.13	GR	34.98	2001	7	M
Rongen et al. [31]	2020	I	0.00	GB	12.98	2001	7	M
Walsh et al. [32]	2020	A	100.00	US	21.50	1993	10	r
Roethlisberger et al. [33]	2020	I	100.00	US	11.70	1993	10	M, r
Samuel et al. [34]	2020	E	56.60	IT	25.17	2001	7	M
Hadiyan and Cosh [35]	2019	E	NR	IR	21.25–24.50	1993	10	M
Kola-Palmer et al. [36]	2019	E	0.00	GB	25.01–25.75	2001	7	M
Pummell and Lavallee [37]	2019	E	42.86	GB	15.10	2001	7	M
Piatt et al. [38]	2018	I	27.66	US	15.57	2001	7	M
Voelker et al. [39]	2018	E	0.00	US	18.45	2001	7	M, r
Bell et al. [40]	2018	A	75.00	US	20.00	2001	7	M
Franck et al. [41]	2018	I	36.36	SE	16.10–16.60	1993	10	M
Chang et al. [42]	2018	A	NR	TW	19.97	2001	7	M, r
Prioios 2017 [43]	2018	I	30.20	GR	19.78	1993	10	r
Giannone et al. [44]	2017	A	75.00	CA	22.10	2001	7	M, r
O’Rourke et al. [45]	2017	I	52.94	US	14.53	2001	7	M, r
Turton et al. [46]	2017	I	44.31	GB	38.77	2001	7	M, r
Geukes et al. [47]	2017	E	49.07	AU	24.06	2001	7	M
Fuller [48]	2017	A	0.00	US	20.00	2001	7	M, r
Van Lone et al. [49]	2017	M	48.00	US	20.00	1993	10	M
Peiró-Velert et al. [50]	2016	Y	49.43	ES	15.00	2001	7	M
Nagata and Long [51]	2016	A	8.62	US	34.79	1993	10	M
Haralabos et al. [52]	2016	A	40.00	GR	18.50	1993	10	M, r
Samuel et al. [53]	2016	E	0.00	IL	31.81	2001	7	M
Franck et al. [54]	2016	I	31.12	SE	16.27, 16.44, 16.69	1993	10	M
Huang et al. [55]	2016	A	35.00	TW	21.58	2001	7	M, r
Waldron [56]	2015	A	65.16	US	17.80	1993	10	M, r
Samuel et al. [57]	2015	E	0.00	US	21.83	2001	7	M
Poux and Fry [58]	2015	A	50.00	US	20.00	1993	7	M, r
Houle and Kluck [59]	2015	A	52.00	US	20.00	2001	7	M
Schutte and McNeil [60]	2015	R	50.50	US, AU	45.95	1993	10	M, r
de Subijana et al. [61]	2015	E	50.00	ES	21.4, 22.4	1993	10	M
Kroshus et al. [62]	2015	A	0.00	US	20.60	2001	7	M
Stambulova et al. [63]	2015	A	50.00	SE	16.00	1993	10	M
Hagiwara and Isogai [64]	2014	M	NR	JP	19.58	2001	7	M
Nagata [65]	2014	A	8.62	US	34.79	1993	10	r
Harris and Watson [66]	2014	M	NR	US	9.17, 12.61, 16	2001	7	M
Price et al. [67]	2014	A	52.00	US	20.53	1993	10	M
Voelker et al. [68]	2014	M	100.00	US	15.63	1993	10	M, r
Madrigal and Gill [69]	2014	A	100.00	US	NR	1993	10	M
Mitchell et al. [70]	2014	Y	NR	GB	17.00	2001	7	M
Bimper [71]	2014	A	0.00	US	19.45	2001	7	M
Poczwardowski et al. [72]	2014	A	33.33	US	23.50	2001	7	M
Petrie et al. [73]	2014	A	0.00	US	20.08	2001	7	M, r
Martin et al. [74]	2014	E	72.58	US	21.61	1993	10	r
Weinberg et al. [75]	2013	R	47.69	US	20.03	1993	10	M
Proios [76]	2013	Y	79.29	GR	11.86	2001	7	M, r
Tyrance et al. [77]	2013	A	52.60	US	20.07	2001	7	M
Martin and Horn [78]	2013	I	100.00	US	16.64	2001	7	M, r
McKay et al. [79]	2013	E	0.00	CA	15.00	1993	10	M
Tasiemski et al. [80]	2013	M	24.14	PL	35.00	2001	7	M
Verkooijen et al. [81]	2012	A	51.00	NL	18.60, 19.20	2001	7	M
Wiśniowska et al. [82]	2012	A	0.00	PL	24,35	2001	7	M
Tasiemski et al. [83]	2012	E	28.00	PL	33,37	2001	7	M
Steinfeldt and Steinfeldt [84]	2012	A	0.00	US	19.53	2001	7	M
Harrison et al. [85]	2011	A	0.00	US	NR	1993	10	M
Kissinger et al. [86]	2011	A	0.00	US	NR	1993	10	M
Samuel and Tenenbaum [87]	2011	M	37.28	US	21.20	2001	7	M
Sturm et al. [88]	2011	A	35.64	US	20.00	1993	10	M, r
Steinfeldt et al. [89]	2011	A	0.00	US	19.39	2001	7	r
Gapin and Petruzello [90]	2011	R	50.84	US	35.88	1993	10	M, r
Visek et al. [91]	2010	A	0.00	US, CN	20.00	2001	7	M, r
Chen et al. [92]	2010	A	37.50	US	20.00	1993	10	M
Lau et al. [93]	2010	Y	48.69	CN	10 to 12	1993	10	M
Mateos et al. [94]	2010	E	51.43	SI	21.63	1993	10	M, r
Packard [95]	2010	E	51.57	ES	21.40	1993	10	M
Caudroit et al. [96]	2010	I	0.00	FR	23.00	1993	10	M, r
Steinfeldt and Steinfeldt [97]	2010	I	0.00	US	15.74	2001	7	M
Steinfeldt et al. [98]	2010	A	0.00	US	19.70	1993	10	r
Maxwell and Visek [99]	2009	M	0.00	CN	NR	2001	7	M, r
Groff et al. [100]	2009	M	50.00	US	18.00	1993	10	M
Kokaridas et al. [101]	2009	E	0.00	GR	30.20	1993	10	r
Steinfeldt et al. [102]	2009	A	0.00	US	19.47	2001	7	M, r
Mateos et al. [103]	2008	E	51.43	SI	21.63	1993	10	M, r
Mignano et al. [104]	2006	A	100.00	US	19.28	2001	7	M
Phoenix et al. [105]	2005	M	49.16	UK, CA	20.00	1993	10	M, r
Albion and Fogarty [106]	2005	A	50.00	AU	16.50	1993	10	M, r
Lau et al. [107]	2004	Y	50.00	GB	12.54	1993	10	M, r
Tasiemskie et al. [108]	2004	M	38.69	GB	44.50	2001	7	M
Grove et al. [109]	2004	A	100.00	AU	16.83	1993	10	M
Schmid and Seiler [110]	2003	E	NR	DE	25.30	2001	7	M, r
Kornspan and Etzel [111]	2001	A	31.27	US	19.38	1993	10	M, r
Horton and Mack [112]	2000	R	25.42	US	40.81	1993	10	M, r
Martin [113] ^	1999	E	47.37	GB, IE, US	16.20	1993	9	M, r
Lantz and Shroeder [114]	1999	A	48.38	US	20.00	1993	10	M
Hale et al. [115]	1999	E	44.62	GB, US	18.32, 19.59	1993	10	M
Smith et al. [116]	1998	M	26.79	GB	32.70	1993	10	r
Wiechman and Williams [117]	1997	I	56.00	US	15.00	1993	10	M
Murphy et al. [118]	1996	A	40.05	US	NR	1993	10	M
Martin et al. ^#^ [6]	1995	E	47.37	GB, IE, US	16.20	1993	9	M, r
Cornelius [119]	1995	R	54.77	US	20.08	1993	10	M, r
Brewer et al. study 1 [4]	1993	M	50.00	US	NR	1993	10	M, r
Brewer et al. study 3 [4]	1993	I	0.00	US	NR	1993	10	M, r

Abbreviations: Bold country abbreviation = study written in non-English language. Level abbreviations: A = advanced, E = elite, I = intermediate, M = mixed, R = recreational, Y = youth. Country abbreviations from https://www.nationsonline.org/oneworld/country_code_list.htm (accessed on 15 June 2022): Australia (AU), Canada (CA), China (CN), Finland (FI), Germany (DE), Ireland (IE), Israel (IL), Italy (IT), Iran (IR), France (FR), Greece (GR), Japan (JP), Poland (PL), Slovenia (SI), Spain (ES), Sweden (SE), Taiwan (TW), The Netherlands (NL), United Kingdom (GB), United States of America (US); Age abbreviation: NR = not reported. Data abbreviations: M = study provided mean AIMS data, r = study provided correlation data. ^ = repeated participants with non-repeating data presented and entered. ^#^ = We chose this Martin et al. [6] study, as it contained more information (mean level and correlates) than the other Martin et al. [5] study with the same participants.

**Table 4:** Summary effect size, heterogeneity, and publication bias statistics for AIMS total and subscale scores.

	Effect Size Statistics						Publication Bias Statistics		
Group	*k*	*n*	*M (SE)*	95% CI	Z-value	*I* ^2^	Fail-Safe *n*	Trim *n*	*M* [95% CI]
AIMS	165	20,498	4.77 (0.08)	4.62, 4.92	62.49	99.62	>1000	19	4.61 [4.47, 4.76]
Exclusivity	22	3976	4.53 (0.20)	4.12, 4.93	21.90	98.55	>1000	0	No adjustment
Neg. Affectivity	21	3916	4.92 (0.21)	4.52, 5.33	23.79	98.63	>1000	4	4.70 [4.28, 5.12]
Self-Identity	4	1187	4.13 (0.11)	3.92, 4.33	38.98	72.97	>1000	1	4.07 [3.86, 4.27]
Social Identity	23	4116	5.24 (0.15)	4.94, 5.53	34.62	98.32	>1000	2	5.14 [4.80, 5.47]

Note: All Z-value *p* < 0.001; Abbreviations: Neg = Negative, *k* = number of samples, *n* = number of participants, *M* = mean, *SE* = standard error, CI = confidence interval, *I*^2^ = heterogeneity statistic.

**Table 5:** Athlete-level summary mixed-effects statistics for AIMS total and subscale scores.

Scale	Category	*k*	*n*	*M* (*SE*)	95% CI	Q_TB_	*p*-Value
AIMS	Elite	38	3209	5.13 (0.15)	4.83, 5.42		
	Advanced	64	8687	5.01 (0.11)	4.78, 5.23		
	Intermediate	29	3466	4.94 (0.17)	4.60, 5.27		
	Recreational	15	2343	3.49 (0.24)	3.02, 3.95		
	Youth	10	1290	3.32 (0.29)	2.75, 3.89	64.09	<0.001

Abbreviations: *k* = number of samples, *n* = number of participants, *M* = mean, *SE* = standard error, CI = confidence interval, Q_TB_ = Q total between statistic.

**Table 6:** Random effects size and publication bias statistics for positive (desirable) and negative (undesirable) correlates with AIMS total and subscales.

	Effect Size Statistics						Publication Bias Statistics			
Correlate	*k*	*n*	*r*	95% CI	Z	*I^2^*	Fail-Safe *n*	Orwin’s *n*	Trim *n*	Mean [95% CI]
*Positive correlates with*										
AIMS	52	6901	0.22	0.12, 0.31	4.46 **	96.41	>1000	109	12R	0.31 [0.22, 0.38]
Exclusivity	12	2902	0.19	−0.10, 0.44	1.30	98.48	961	29	3L	0.05 [−0.31, 0.41]
Neg. Affectivity	13	3115	0.16	−0.06, 0.35	1.44	97.59	638	26	4R	0.26 [0.10, 0.41]
Self-Identity	2	57	0.37	0.16, 0.55	3.37 **	31.90	Not enough data to run analysis			
Social Identity	11	2845	0.31	0.03, 0.54	2.17 *	98.51	>1000	43	2R	0.36 [0.14, 0.54]
*Negative correlates with*										
AIMS	45	3318	0.17	0.11, 0.22	5.69 **	83.46	>1000	44	0	No adjustment
Exclusivity	10	472	0.04	−0.10, 0.18	0.57	83.62	0	0	0	No adjustment
Neg. Affectivity	10	763	0.10	−0.03, 0.22	1.52	82.61	0	0	0	No adjustment
Self-Identity	5	236	−0.06	−0.25, 0.13	−0.61	81.91	0	0	0	No adjustment
Social Identity	10	519	−0.02	−0.16, 0.12	−0.30	83.51	0	0	2L	−0.09 [−0.22, 0.05]

Note: ** Z-value *p* ≤ 0.001; * Z-value *p* < 0.05; Abbreviations: Neg = Negative, *k* = number of samples, *n* = total unique participants, *r* = correlation, CI = confidence interval, *I*^2^ = heterogeneity statistic.

**Table 7:** Random effects size and publication bias statistics for AIMS total score and correlate subcategories.

		Effect Size Statistics					
Factor Category	Correlate	*k*	*n*	*r*	95% CI	Z-Value	*I* ^2^
Positive	Intrinsic motivation, commitment	6	2442	0.51	0.24, 0.71	3.50 **	97.66
Positive	Mastery, task goal orientation	4	761	0.39	0.25, 0.51	5.13 **	72.35
Positive	Introjected, identified regulations	3	492	0.35	0.24, 0.44	6.13 **	61.23
Negative	Ego, win goal orientation	4	761	0.31	0.21, 0.41	5.67 **	47.12
Negative	Amotivation, external regulation	4	562	0.18	−0.08, 0.42	1.36	87.86
Positive	Positive affect, emotions, feelings	16	2486	0.16	0.07, 0.24	3.62 **	81.47
Negative	Neg. affect, emotions, feelings	17	2012	0.16	0.06, 0.27	2.97 *	84.92
Negative	Body issues	20	1116	0.14	0.11, 0.17	8.39 **	47.12
Positive	Self-esteem, worth, competence	23	3645	0.13	0.04, 0.21	2.97 *	86.09

Note: ** Z-value *p* ≤ 0.001; * Z-value *p* < 0.05; Abbreviations: Neg = Negative, *k* = number of samples, *n* = total unique participants, *r* = correlation, CI = confidence interval, *I*^2^ = heterogeneity statistic.

## Data Availability

All data are contained in the article tables and Supplementary Materials.

## Supplementary Materials

The following supporting information can be downloaded at: https://www.mdpi.com/article/10.3390/ejihpe12090097/s1, Table S1: PRISMA 2020 checklist; Table S2: Study-level AIMS and AIMS subscale effect size statistics; Table S3: Individual study correlation statistics and information.
