# Supplementary material for: The Athletic Identity Measurement Scale: A Systematic Review with Meta-Analysis from 1993 to 2021
Source: Eur J Investig Health Psychol Educ. 2022 Sep 14;12(9):1391–414. doi: 10.3390/ejihpe12090097 (PMC9497853; doi:10.3390/ejihpe12090097)
Supplement: Supplementary file 1 [file ejihpe-12-00097-s001.zip › ejihpe-1839975-supplementary.pdf]

Supplement Files

Table S1. PRISMA 2020 checklist.

Section and Topic	Item #	Checklist item	Location where item is reported
**TITLE**			
Title	1	Identify the report as a systematic review.	Lines 2–3
**ABSTRACT**			
Abstract	2	See the PRISMA 2020 for Abstracts checklist.	Lines 12–32 with MDPI word limit.
**INTRODUCTION**			
Rationale	3	Describe the rationale for the review in the context of existing knowledge.	1. Introduction
Objectives	4	Provide an explicit statement of the objective(s) or question(s) the review addresses.	1.2. Research Questions
**METHODS**			
Eligibility criteria	5	Specify the inclusion and exclusion criteria for the review and how studies were grouped for the syntheses.	2.1. Eligibility
Information sources	6	Specify all databases, registers, websites, organizations, reference lists and other sources searched or consulted to identify studies. Specify the date when each source was last searched or consulted.	2.2. Information Sources and Search Strategy
Search strategy	7	Present the full search strategies for all databases, registers, and websites, including any filters and limits used.	Presented an example, as our method is simple to follow.
Selection process	8	Specify the methods used to decide whether a study met the inclusion criteria of the review, including how many reviewers screened each record and each report retrieved, whether they worked independently, and if applicable, details of automation tools used in the process.	2.3. Article and Data Selection Process and Data Items
Data collection process	9	Specify the methods used to collect data from reports, including how many reviewers collected data from each report, whether they worked independently, any processes for obtaining or confirming data from study investigators, and if applicable, details of automation tools used in the process.	2.3.
Data items	10a	List and define all outcomes for which data were sought. Specify whether all results that were compatible with each outcome domain in each study were sought (e.g., for all measures, time points, and analyses), and if not, the methods used to decide which results to collect.	2.3.
	10b	List and define all other variables for which data were sought (e.g., participant and intervention characteristics and funding sources). Describe any assumptions made about any missing or unclear information.	2.3.
Study risk of bias assessment	11	Specify the methods used to assess the risk of bias in the included studies, including details of the tool(s) used, how many reviewers assessed each study and whether they worked independently, and if applicable, details of automation tools used in the process.	2.4. Risk of Bias Assessments
Effect measures	12	Specify for each outcome the effect measure(s) (e.g., risk ratio and mean difference) used in the synthesis or presentation of results.	2.5. Effect Size Measures, Synthesis Methods, and Certainty Assessment
Synthesis	13a	Describe the processes used to decide which studies were eligible for each synthesis (e.g., tabulating the study intervention	2.5.

Supplement Files

Section and Topic	Item #	Checklist item	Location where item is reported
methods		characteristics and comparing against the planned groups for each synthesis (item #5)).	
	13b	Describe any methods required to prepare the data for presentation or synthesis, such as the handling of missing summary statistics or data conversions.	2.5.
	13c	Describe any methods used to tabulate or visually display the results of individual studies and syntheses.	2.5.
	13d	Describe any methods used to synthesize results and provide a rationale for the choice(s). If a meta-analysis was performed, describe the model(s), method(s) to identify the presence and extent of statistical heterogeneity, and software package(s) used.	2.5.
	13e	Describe any methods used to explore possible causes of heterogeneity among study results (e.g., subgroup analysis and meta-regression).	2.5.
	13f	Describe any sensitivity analyses conducted to assess the robustness of the synthesized results.	Orwin's N somewhat addresses this.
Reporting bias assessment	14	Describe any methods used to assess the risk of bias due to missing results in a synthesis (arising from reporting biases).	2.4.
Certainty assessment	15	Describe any methods used to assess certainty (or confidence) in the body of evidence for an outcome.	2.5.
		**RESULTS**	
Study selection	16a	Describe the results of the search and selection process, from the number of records identified in the search to the number of studies included in the review, ideally using a flow diagram.	3.1. Study Selection and Characteristics
	16b	Cite studies that might appear to meet the inclusion criteria but were excluded, and explain why they were excluded.	Available from ML
Study characteristics	17	Cite each included study and present its characteristics.	Table 2
Risk of bias in studies	18	Present assessments of risk of bias for each included study.	Table 3, 3.2. Risk of Bias within Studies
Results of individual studies	19	For all outcomes, present, for each study: (a) summary statistics for each group (where appropriate) and (b) an effect estimate and its precision (e.g., confidence/credible interval), ideally using structured tables or plots.	See results for mean level and correlations
Results of syntheses	20a	For each synthesis, briefly summarize the characteristics and the risk of bias among the contributing studies.	See result tables for mean level and correlates.
	20b	Present the results of all statistical syntheses conducted. If a meta-analysis was performed, present for each the summary estimate and its precision (e.g., confidence/credible interval) and measures of statistical heterogeneity. If comparing groups, describe the direction of the effect.	See results for mean level and correlations
	20c	Present the results of all investigations of possible causes of heterogeneity among the study results.	The mixed-effect analyses somewhat address the possible causes—groups of athletes within the larger total sample and subgroups

Supplement Files

Section and Topic	Item #	Checklist item	Location where item is reported
			of positive and negative factors.
	20d	Present the results of all sensitivity analyses conducted to assess the robustness of the synthesized results.	Orwin's N somewhat addresses this.
Reporting biases	21	Present the assessments of the risk of bias due to missing results (arising from reporting biases) for each synthesis assessed.	See all funnel plot figures
Certainty of evidence	22	Present the assessments of certainty (or confidence) in the body of evidence for each outcome assessed.	Moved to discussion section based on reviewer comment.
**DISCUSSION**			
Discussion	23a	Provide a general interpretation of the results in the context of other evidence.	4. Discussion
	23b	Discuss any limitations of the evidence included in the review.	4.1. AIMS Literature and Study Limitations
	23c	Discuss any limitations of the review processes used.	4.1.
	23d	Discuss the implications of the results for practice, policy, and future research.	Presented across discussion
**OTHER INFORMATION**			
Registration and protocol	24a	Provide registration information for the review, including the register name and registration number, or state that the review was not registered.	None
	24b	Indicate where the review protocol can be accessed, or state that a protocol was not prepared.	See end materials
	24c	Describe and explain any amendments to information provided at registration or in the protocol.	None
Support	25	Describe sources of financial or non-financial support for the review and the role of the funders or sponsors in the review.	See end materials
Competing interests	26	Declare any competing interests of review authors.	None, see end materials
Availability of data, code, and other materials	27	Report which of the following are publicly available and where they can be found: template data collection forms; data extracted from included studies; data used for all analyses; analytic code; any other materials used in the review.	Reported ML has the documents.

From: Page, M.J.; McKenzie, J.E.; Bossuyt, P.M.; Boutron, I.; Hoffmann, T.C.; Mulrow, C.D.; Shamseer, L.; Tetzlaff, J.M.; Akl, E.A.; Brennan, S.E.; et al. The PRISMA 2020 statement: an updated guideline for reporting systematic reviews. *BMJ* **2021**, *372*, n71. https://doi.org/10.1136/bmj.n71.

For more information, visit: http://www.prisma-statement.org/

Supplement Files

Table S2. Study-level AIMS and AIMS subscale effect size statistics.

			Statistics for each study						
**Scale**	**Authors, publication year**	**[Ref#]**	**Mean**	**SE**	**Variance**	**95% CI LL**	**95% CI UL**	**Z-Value**	***p*-Value**
AIMS Total	Albion and Fogarty 2005	[106]	3.74	0.08	0.01	3.58	3.90	44.83	0.00
AIMS Total	Albion and Fogarty 2005	[106]	3.74	0.01	0.00	3.73	3.75	657.49	0.00
AIMS Total	Bell et al. 2018	[40]	5.51	0.19	0.03	5.14	5.88	29.48	0.00
AIMS Total	Bell et al. 2018	[40]	4.10	0.27	0.07	3.57	4.63	15.24	0.00
AIMS Total	Bell et al. 2018	[40]	6.21	0.11	0.01	6.00	6.42	58.60	0.00
AIMS Total	Bimper 2014	[71]	5.67	0.02	0.00	5.63	5.71	274.37	0.00
AIMS Total	Brewer et al. 1993	[4]	3.48	0.12	0.01	3.24	3.72	28.77	0.00
AIMS Total	Brewer et al. 1993	[4]	3.04	0.13	0.02	2.79	3.29	24.25	0.00
AIMS Total	Brewer et al. 1993	[4]	4.68	0.17	0.03	4.35	5.01	27.57	0.00
AIMS Total	Brewer et al. 1993	[4]	4.04	0.34	0.12	3.37	4.71	11.88	0.00
AIMS Total	Brewer et al. 1993	[4]	5.46	0.18	0.03	5.12	5.80	31.18	0.00
AIMS Total	Brewer et al. 1993	[4]	5.34	0.22	0.05	4.91	5.77	24.19	0.00
AIMS Total	Caudroit et al. 2010	[96]	4.66	0.09	0.01	4.47	4.85	49.29	0.00
AIMS Total	Chang et al. 2018	[42]	4.25	0.05	0.00	4.14	4.36	78.76	0.00
AIMS Total	Chen et al. 2010	[92]	3.81	0.05	0.00	3.72	3.90	83.13	0.00
AIMS Total	Cornelius 1995	[119]	3.31	0.08	0.01	3.15	3.47	39.95	0.00
AIMS Total	Cornelius 1995	[119]	3.55	0.12	0.01	3.32	3.78	29.95	0.00
AIMS Total	Cornelius 1995	[119]	3.18	0.11	0.01	2.96	3.40	28.91	0.00
AIMS Total	Costa et al. 2020	[26]	5.27	0.07	0.01	5.13	5.41	71.96	0.00
AIMS Total	de Subijana et al. 2015	[61]	5.41	0.14	0.02	5.13	5.69	37.48	0.00
AIMS Total	de Subijana et al. 2015	[61]	5.75	0.07	0.00	5.61	5.89	82.14	0.00
AIMS Total	Franck et al. 2016	[54]	6.57	0.10	0.01	6.38	6.76	66.08	0.00
AIMS Total	Franck et al. 2016	[54]	5.41	0.11	0.01	5.20	5.62	49.34	0.00
AIMS Total	Franck et al. 2016	[54]	4.26	0.16	0.02	3.95	4.57	27.10	0.00
AIMS Total	Franck et al. 2018	[41]	5.73	0.28	0.08	5.19	6.27	20.66	0.00
AIMS Total	Franck et al. 2018	[41]	5.19	0.26	0.07	4.69	5.69	20.26	0.00
AIMS Total	Franck et al. 2018	[41]	5.79	0.10	0.01	5.59	5.99	56.29	0.00
AIMS Total	Gapin and Petruzello 2011	[90]	4.26	0.09	0.01	4.08	4.44	46.45	0.00
AIMS Total	Gapin and Petruzello 2011	[90]	3.47	0.09	0.01	3.30	3.64	39.80	0.00
AIMS Total	Geary et al. 2021	[23]	5.16	0.17	0.03	4.83	5.49	30.35	0.00
AIMS Total	Geukes et al. 2017	[47]	6.43	0.08	0.01	6.28	6.58	84.65	0.00

Supplement Files

AIMS Total	Giannone et al. 2017	[44]	3.75	0.07	0.01	3.61	3.89	51.32	0.00
AIMS Total	Graham and Burns 2020	[25]	5.50	0.07	0.01	5.36	5.64	75.09	0.00
AIMS Total	Graupensperger et al. 2020	[29]	6.13	0.06	0.00	6.02	6.24	109.04	0.00
AIMS Total	Groff et al. 2009	[100]	4.86	0.13	0.02	4.61	5.11	37.59	0.00
AIMS Total	Groff et al. 2009	[100]	4.94	0.24	0.06	4.47	5.41	20.48	0.00
AIMS Total	Groff et al. 2009	[100]	5.46	0.18	0.03	5.12	5.80	31.18	0.00
AIMS Total	Groff et al. 2009	[100]	5.34	0.22	0.05	4.91	5.77	24.19	0.00
AIMS Total	Groff et al. 2009	[100]	4.80	0.37	0.14	4.07	5.53	12.92	0.00
AIMS Total	Groff et al. 2009	[100]	3.48	0.12	0.01	3.24	3.72	28.77	0.00
AIMS Total	Groff et al. 2009	[100]	3.04	0.13	0.02	2.79	3.29	24.25	0.00
AIMS Total	Groff et al. 2009	[100]	3.92	0.12	0.02	3.68	4.16	31.46	0.00
AIMS Total	Groff et al. 2009	[100]	3.51	0.15	0.02	3.22	3.80	23.78	0.00
AIMS Total	Groff et al. 2009	[100]	4.43	0.21	0.04	4.02	4.84	21.10	0.00
AIMS Total	Groff et al. 2009	[100]	4.57	0.23	0.06	4.11	5.03	19.46	0.00
AIMS Total	Groff et al. 2009	[100]	4.81	0.20	0.04	4.42	5.20	24.00	0.00
AIMS Total	Grove et al. 2004	[109]	4.69	0.14	0.02	4.43	4.95	34.71	0.00
AIMS Total	Hadiyan and Cosh 2019	[64]	5.38	0.06	0.00	5.26	5.50	84.54	0.00
AIMS Total	Hadiyan and Cosh 2019	[64]	5.35	0.10	0.01	5.16	5.54	55.36	0.00
AIMS Total	Hagiwara and Isogai 2014	[64]	5.64	0.05	0.00	5.54	5.74	109.09	0.00
AIMS Total	Hagiwara and Isogai 2014	[64]	5.30	0.07	0.00	5.16	5.44	74.96	0.00
AIMS Total	Hagiwara et al. 2020	[24]	4.70	0.03	0.00	4.63	4.77	138.18	0.00
AIMS Total	Hagiwara et al. 2020	[24]	3.22	0.06	0.00	3.10	3.34	51.88	0.00
AIMS Total	Hale et al. 1999	[115]	5.20	0.08	0.01	5.04	5.36	65.67	0.00
AIMS Total	Hale et al. 1999	[115]	4.58	0.05	0.00	4.48	4.68	85.52	0.00
AIMS Total	Hale et al. 1999	[115]	4.81	0.11	0.01	4.59	5.03	42.36	0.00
AIMS Total	Haralabos et al. 2016	[52]	4.87	0.08	0.01	4.71	5.03	59.65	0.00
AIMS Total	Harris and Watson 2014	[66]	3.52	0.13	0.02	3.27	3.77	28.11	0.00
AIMS Total	Harris and Watson 2014	[66]	3.44	0.07	0.00	3.31	3.57	50.93	0.00
AIMS Total	Harris and Watson 2014	[66]	5.11	0.13	0.02	4.86	5.36	40.33	0.00
AIMS Total	Harrison et al. 2011	[85]	3.44	0.10	0.01	3.24	3.64	34.22	0.00
AIMS Total	Horton and Mack 2000	[112]	4.09	0.06	0.00	3.97	4.21	67.56	0.00
AIMS Total	Houle and Kluck 2015	[59]	5.37	0.07	0.00	5.24	5.50	82.30	0.00
AIMS Total	Huang et al. 2016	[55]	4.07	0.03	0.00	4.01	4.13	132.63	0.00
AIMS Total	Ioannis 2020	[30]	4.60	0.13	0.02	4.35	4.85	36.47	0.00

Supplement Files

AIMS Total	Kissinger et al. 2011	[86]	4.90	0.07	0.00	4.77	5.03	74.49	0.00
AIMS Total	Kissinger et al. 2011	[86]	5.90	0.05	0.00	5.80	6.00	120.80	0.00
AIMS Total	Kissinger et al. 2011	[86]	6.10	0.05	0.00	6.00	6.20	120.83	0.00
AIMS Total	Kola-Palmer et al. 2019	[36]	5.38	0.10	0.01	5.19	5.57	54.89	0.00
AIMS Total	Kola-Palmer et al. 2019	[36]	5.36	0.07	0.00	5.23	5.49	78.29	0.00
AIMS Total	Koper et al. 2020	[27]	5.39	0.24	0.06	4.92	5.86	22.64	0.00
AIMS Total	Koper et al. 2020	[27]	5.42	0.22	0.05	4.99	5.85	24.86	0.00
AIMS Total	Koper et al. 2020	[27]	5.24	0.22	0.05	4.82	5.66	24.26	0.00
AIMS Total	Koper et al. 2020	[27]	5.60	0.22	0.05	5.17	6.03	25.64	0.00
AIMS Total	Kornspan and Etzel 2001	[111]	5.01	0.07	0.00	4.88	5.14	75.35	0.00
AIMS Total	Kroshus et al. 2015	[62]	5.68	0.06	0.00	5.56	5.80	89.96	0.00
AIMS Total	Lantz and Shroeder 1999	[114]	5.03	0.08	0.01	4.88	5.18	63.65	0.00
AIMS Total	Lantz and Shroeder 1999	[114]	5.04	0.11	0.01	4.83	5.25	46.69	0.00
AIMS Total	Lantz and Shroeder 1999	[114]	5.02	0.12	0.01	4.79	5.25	42.18	0.00
AIMS Total	Lau et al. 2004	[107]	2.74	0.07	0.00	2.61	2.87	41.06	0.00
AIMS Total	Lau et al. 2004	[107]	2.94	0.06	0.00	2.82	3.06	47.80	0.00
AIMS Total	Lau et al. 2004	[107]	2.57	0.06	0.00	2.45	2.69	42.50	0.00
AIMS Total	Lau et al. 2010	[93]	3.13	0.04	0.00	3.04	3.22	70.06	0.00
AIMS Total	Lau et al. 2010	[93]	3.26	0.06	0.00	3.13	3.39	50.88	0.00
AIMS Total	Lau et al. 2010	[93]	3.00	0.06	0.00	2.88	3.12	48.86	0.00
AIMS Total	Lau et al. 2010	[93]	3.13	0.05	0.00	3.03	3.23	61.48	0.00
AIMS Total	Lau et al. 2010	[93]	3.14	0.10	0.01	2.95	3.33	32.53	0.00
AIMS Total	Madrigal and Gill 2014	[69]	5.42	0.39	0.15	4.66	6.18	13.90	0.00
AIMS Total	Martin and Horn 2013	[78]	5.46	0.09	0.01	5.29	5.63	62.05	0.00
AIMS Total	Martin 1999	[113]	4.12	0.16	0.02	3.81	4.43	26.36	0.00
AIMS Total	Mateos et al. 2008	[103]	5.38	0.14	0.02	5.11	5.65	39.79	0.00
AIMS Total	Mateos et al. 2010	[94]	5.38	0.14	0.02	5.11	5.65	39.79	0.00
AIMS Total	Maxwell and Visek 2009	[99]	4.92	0.13	0.02	4.67	5.17	39.05	0.00
AIMS Total	Maxwell and Visek 2009	[99]	4.91	0.11	0.01	4.70	5.12	46.21	0.00
AIMS Total	McKay et al. 2013	[79]	5.56	0.04	0.00	5.48	5.64	131.78	0.00
AIMS Total	Mignano et al. 2006	[104]	5.32	0.09	0.01	5.15	5.49	60.19	0.00
AIMS Total	Mignano et al. 2006	[104]	4.80	0.13	0.02	4.54	5.06	36.37	0.00
AIMS Total	Mignano et al. 2006	[104]	5.57	0.09	0.01	5.39	5.75	59.83	0.00
AIMS Total	Mignano et al. 2006	[104]	5.97	0.09	0.01	5.80	6.14	68.93	0.00

Supplement Files

AIMS Total	Mignano et al. 2006	[104]	4.93	0.13	0.02	4.68	5.18	38.29	0.00
AIMS Total	Mignano et al. 2006	[104]	4.84	0.08	0.01	4.67	5.01	57.43	0.00
AIMS Total	Mignano et al. 2006	[104]	4.73	0.16	0.03	4.41	5.05	29.07	0.00
AIMS Total	Mignano et al. 2006	[104]	5.03	0.11	0.01	4.81	5.25	45.24	0.00
AIMS Total	Mitchell et al. 2014	[70]	5.79	0.06	0.00	5.68	5.90	101.41	0.00
AIMS Total	Moazami-Goodarzi et al. 2020	[28]	5.17	0.04	0.00	5.09	5.25	132.77	0.00
AIMS Total	Murphy et al. 1996	[118]	4.96	0.09	0.01	4.78	5.14	54.15	0.00
AIMS Total	Murphy et al. 1996	[118]	5.05	0.09	0.01	4.86	5.24	53.45	0.00
AIMS Total	Murphy et al. 1996	[118]	4.60	0.25	0.06	4.10	5.10	18.17	0.00
AIMS Total	Nagata and Long 2016	[51]	4.46	0.22	0.05	4.03	4.89	20.28	0.00
AIMS Total	Ohji et al. 2021	[22]	5.71	0.24	0.06	5.24	6.18	24.02	0.00
AIMS Total	O'Rourke et al. 2017	[45]	3.83	0.09	0.01	3.66	4.00	44.12	0.00
AIMS Total	Packard 2010	[95]	4.23	0.14	0.02	3.95	4.51	29.37	0.00
AIMS Total	Packard 2010	[95]	4.53	0.09	0.01	4.36	4.70	52.11	0.00
AIMS Total	Packard 2010	[95]	4.12	0.23	0.05	3.67	4.57	18.07	0.00
AIMS Total	Petrie et al. 2014	[73]	4.60	0.09	0.01	4.43	4.77	53.81	0.00
AIMS Total	Piatt et al. 2018	[38]	5.34	0.03	0.00	5.29	5.39	207.58	0.00
AIMS Total	Piatt et al. 2018	[38]	5.67	0.07	0.00	5.53	5.81	81.77	0.00
AIMS Total	Poczwardowski et al. 2014	[72]	5.29	0.29	0.08	4.72	5.86	18.25	0.00
AIMS Total	Poux and Fry 2015	[58]	5.76	0.08	0.01	5.60	5.92	71.11	0.00
AIMS Total	Price et al. 2014	[67]	5.03	0.08	0.01	4.88	5.18	66.16	0.00
AIMS Total	Pummell and Lavallee 2019	[37]	5.51	0.23	0.05	5.06	5.96	23.90	0.00
AIMS Total	Roethlisberger 2020	[33]	5.08	0.10	0.01	4.89	5.27	52.18	0.00
AIMS Total	Rongen 2020	[31]	5.75	0.11	0.01	5.53	5.97	50.82	0.00
AIMS Total	Samuel and Tenebaum 2011	[87]	5.55	0.05	0.00	5.45	5.65	105.18	0.00
AIMS Total	Samuel 2020	[34]	5.81	0.11	0.01	5.59	6.03	52.87	0.00
AIMS Total	Samuel et al. 2015	[57]	6.53	0.15	0.02	6.25	6.81	45.03	0.00
AIMS Total	Samuel et al. 2016	[53]	5.62	0.15	0.02	5.33	5.91	37.81	0.00
AIMS Total	Schmid and Seiler 2003	[110]	5.15	0.06	0.00	5.03	5.27	85.94	0.00
AIMS Total	Shutte and McNeil 2015	[60]	2.66	0.07	0.00	2.52	2.80	38.55	0.00
AIMS Total	Stambulova et al. 2015	[63]	5.87	0.05	0.00	5.78	5.96	128.91	0.00
AIMS Total	Steinfeldt and Steinfeldt 2010	[97]	3.79	0.06	0.00	3.68	3.90	66.28	0.00
AIMS Total	Steinfeldt and Steinfeldt 2010	[97]	5.15	0.14	0.02	4.88	5.42	37.50	0.00
AIMS Total	Steinfeldt and Steinfeldt 2010	[97]	5.13	0.13	0.02	4.88	5.38	39.60	0.00

Supplement Files

AIMS Total	Steinfeldt and Steinfeldt 2010	[97]	5.16	0.20	0.04	4.77	5.55	26.25	0.00
AIMS Total	Steinfeldt and Steinfeldt 2010	[97]	4.95	0.17	0.03	4.62	5.28	29.77	0.00
AIMS Total	Steinfeldt and Steinfeldt 2010	[97]	5.09	0.08	0.01	4.94	5.24	66.76	0.00
AIMS Total	Steinfeldt and Steinfeldt 2012	[84]	5.15	0.04	0.00	5.08	5.22	145.40	0.00
AIMS Total	Steinfeldt et al. 2009	[102]	5.16	0.06	0.00	5.05	5.27	88.18	0.00
AIMS Total	Steinfeldt et al. 2009	[102]	5.52	0.07	0.01	5.38	5.66	77.43	0.00
AIMS Total	Steinfeldt et al. 2009	[102]	4.99	0.11	0.01	4.77	5.21	43.65	0.00
AIMS Total	Steinfeldt et al. 2009	[102]	4.66	0.11	0.01	4.45	4.87	43.75	0.00
AIMS Total	Sturm et al. 2011	[88]	4.85	0.10	0.01	4.66	5.04	50.51	0.00
AIMS Total	Sturm et al. 2011	[88]	4.93	0.08	0.01	4.78	5.08	63.32	0.00
AIMS Total	Tasiemski et al. 2012	[83]	3.54	0.32	0.10	2.91	4.17	11.06	0.00
AIMS Total	Tasiemski et al. 2012	[83]	5.20	0.19	0.04	4.83	5.57	27.37	0.00
AIMS Total	Tasiemski et al. 2013	[80]	5.65	0.20	0.04	5.26	6.04	28.71	0.00
AIMS Total	Tasiemski et al. 2013	[80]	5.06	0.20	0.04	4.67	5.45	25.57	0.00
AIMS Total	Tasiemskie et al. 2004	[108]	3.73	0.20	0.04	3.33	4.13	18.37	0.00
AIMS Total	Tasiemskie et al. 2004	[108]	3.07	0.18	0.03	2.72	3.42	17.04	0.00
AIMS Total	Tasiemskie et al. 2004	[108]	2.81	0.16	0.03	2.49	3.13	17.16	0.00
AIMS Total	Turton et al. 2017	[46]	4.57	0.04	0.00	4.49	4.65	115.66	0.00
AIMS Total	Turton et al. 2017	[46]	4.36	0.04	0.00	4.27	4.45	96.96	0.00
AIMS Total	Tyrance et al. 2013	[77]	5.27	0.04	0.00	5.20	5.34	145.52	0.00
AIMS Total	Van Lone et al. 2017	[49]	2.89	0.09	0.01	2.72	3.06	33.93	0.00
AIMS Total	Verkooijen et al. 2012	[81]	5.92	0.15	0.02	5.62	6.22	38.85	0.00
AIMS Total	Verkooijen et al. 2012	[81]	5.41	0.21	0.04	4.99	5.83	25.51	0.00
AIMS Total	Visek et al. 2010	[91]	5.57	0.04	0.00	5.48	5.66	124.68	0.00
AIMS Total	Visek et al. 2010	[91]	5.16	0.06	0.00	5.04	5.28	87.73	0.00
AIMS Total	Voelker et al. 2014	[39]	4.81	0.06	0.00	4.69	4.93	77.02	0.00
AIMS Total	Voelker et al. 2018	[39]	5.68	0.20	0.04	5.28	6.08	28.06	0.00
AIMS Total	Waldron 2015	[56]	4.87	0.03	0.00	4.80	4.94	144.74	0.00
AIMS Total	Weinberg et al. 2013	[75]	4.15	0.11	0.01	3.94	4.36	39.11	0.00
AIMS Total	Wiechman and Williams 1997	[117]	4.97	0.03	0.00	4.90	5.04	146.96	0.00
AIMS Total	Wiśniowska et al. 2012	[82]	4.12	0.39	0.15	3.36	4.88	10.59	0.00
AIMS Total	Wiśniowska et al. 2012	[82]	5.40	0.28	0.08	4.84	5.96	19.02	0.00
Exclusivity	Costa et al. 2020	[26]	4.88	0.07	0.01	4.73	5.03	65.28	0.00
Exclusivity	Fuller 2017	[48]	4.47	0.11	0.01	4.25	4.69	39.15	0.00

Supplement Files

Exclusivity	Gapin and Petruzello 2011	[90]	3.33	0.15	0.02	3.03	3.63	22.09	0.00
Exclusivity	Gapin and Petruzello 2011	[90]	2.30	0.14	0.02	2.03	2.57	16.51	0.00
Exclusivity	Geary et al. 2021	[23]	5.90	0.36	0.13	5.19	6.61	16.39	0.00
Exclusivity	Hagiwara et al. 2020	[24]	5.20	0.05	0.00	5.10	5.30	102.90	0.00
Exclusivity	Hagiwara et al. 2020	[24]	3.44	0.08	0.01	3.29	3.59	44.04	0.00
Exclusivity	Ioannis 2020	[30]	4.35	0.14	0.02	4.08	4.62	31.08	0.00
Exclusivity	Martin and Horn 2013	[78]	4.39	0.11	0.01	4.17	4.61	38.63	0.00
Exclusivity	Martin et al. 1995	[6]	3.57	0.22	0.05	3.15	3.99	16.54	0.00
Exclusivity	Mitchell et al. 2014	[70]	5.75	0.09	0.01	5.58	5.92	64.81	0.00
Exclusivity	Nagata and Long 2016	[51]	3.65	0.21	0.04	3.23	4.07	17.22	0.00
Exclusivity	Peiró-Velert et al. 2016	[50]	4.76	0.09	0.01	4.59	4.93	54.03	0.00
Exclusivity	Phoenix et al. 2005 2	[105]	3.53	0.15	0.02	3.24	3.82	23.78	0.00
Exclusivity	Phoenix et al. 2005 5	[105]	4.19	0.17	0.03	3.86	4.52	24.78	0.00
Exclusivity	Poczwardowski et al. 2014	[72]	4.50	0.50	0.25	3.52	5.48	8.96	0.00
Exclusivity	Rongen 2020	[31]	5.85	0.17	0.03	5.52	6.18	35.01	0.00
Exclusivity	Samuel and Tenebaum 2011	[87]	4.70	0.09	0.01	4.53	4.87	54.12	0.00
Exclusivity	Samuel et al. 2015	[57]	6.87	0.13	0.02	6.63	7.11	54.96	0.00
Exclusivity	Tasiemski et al. 2013	[80]	5.45	0.30	0.09	4.87	6.03	18.33	0.00
Exclusivity	Tasiemski et al. 2013	[80]	5.07	0.21	0.05	4.65	5.49	23.86	0.00
Exclusivity	Weinberg et al. 2013	[75]	3.67	0.14	0.02	3.40	3.94	26.82	0.00
Negative Affectivity	Costa et al. 2020	[26]	5.54	0.08	0.01	5.39	5.69	72.64	0.00
Negative Affectivity	Fuller 2017	[48]	5.56	0.09	0.01	5.38	5.74	60.05	0.00
Negative Affectivity	Gapin and Petruzello 2011	[90]	5.55	0.14	0.02	5.28	5.82	39.89	0.00
Negative Affectivity	Gapin and Petruzello 2011	[90]	4.41	0.16	0.02	4.10	4.72	28.22	0.00
Negative Affectivity	Geary et al. 2021	[23]	5.40	0.49	0.24	4.45	6.35	11.10	0.00
Negative Affectivity	Hagiwara et al. 2020	[24]	5.26	0.05	0.00	5.16	5.36	104.76	0.00
Negative Affectivity	Hagiwara et al. 2020	[24]	3.86	0.08	0.01	3.70	4.02	48.37	0.00
Negative Affectivity	Ioannis 2020	[30]	4.96	0.13	0.02	4.71	5.21	39.06	0.00
Negative Affectivity	Martin and Horn 2013	[78]	5.17	0.10	0.01	4.97	5.37	51.09	0.00
Negative Affectivity	Martin et al. 1995	[6]	5.20	0.23	0.05	4.75	5.65	22.43	0.00
Negative affectivity	Mitchell et al. 2014	[70]	6.15	0.07	0.01	6.01	6.29	83.91	0.00
Negative Affectivity	Nagata and Long 2016	[51]	4.46	0.23	0.05	4.01	4.91	19.35	0.00
Negative Affectivity	Peiró-Velert et al. 2016	[50]	3.43	0.08	0.01	3.27	3.59	41.88	0.00
Negative Affectivity	Phoenix et al. 2005	[105]	2.22	0.16	0.03	1.91	2.53	13.87	0.00

Supplement Files

Negative Affectivity	Phoenix et al. 2005	[105]	5.46	0.16	0.03	5.14	5.78	33.57	0.00
Negative Affectivity	Poczwardowski et al. 2014	[72]	4.84	0.54	0.29	3.78	5.90	8.91	0.00
Negative Affectivity	Rongen 2020	[31]	5.74	0.21	0.05	5.32	6.16	26.81	0.00
Negative Affectivity	Samuel and Tenebaum 2011	[87]	5.77	0.07	0.00	5.64	5.90	83.98	0.00
Negative Affectivity	Tasiemski et al. 2013	[80]	5.75	0.30	0.09	5.15	6.35	18.91	0.00
Negative Affectivity	Tasiemski et al. 2013	[80]	4.67	0.32	0.10	4.05	5.29	14.76	0.00
Negative Affectivity	Weinberg et al. 2013	[75]	4.19	0.12	0.01	3.95	4.43	34.87	0.00
Self-Identity	Gapin and Petruzello 2011	[90]	4.17	0.06	0.00	4.05	4.29	69.40	0.00
Self-Identity	Gapin and Petruzello 2011	[90]	3.87	0.09	0.01	3.69	4.05	42.72	0.00
Self-Identity	Geary et al. 2021	[23]	4.20	0.16	0.02	3.89	4.51	26.81	0.00
Self-Identity	Nagata and Long 2016	[51]	4.42	0.19	0.03	4.05	4.79	23.67	0.00
Social Identity	Costa et al. 2020	[26]	5.35	0.07	0.01	5.21	5.49	75.66	0.00
Social Identity	Fuller 2017	[48]	6.16	0.07	0.00	6.03	6.29	91.77	0.00
Social Identity	Gapin and Petruzello 2011	[90]	4.44	0.15	0.02	4.14	4.74	29.46	0.00
Social Identity	Gapin and Petruzello 2011	[90]	3.69	0.16	0.02	3.39	3.99	23.79	0.00
Social Identity	Geary et al. 2021	[23]	5.30	0.28	0.08	4.75	5.85	18.93	0.00
Social Identity	Hagiwara et al. 2020	[24]	5.71	0.04	0.00	5.63	5.79	144.48	0.00
Social Identity	Hagiwara et al. 2020	[24]	3.88	0.07	0.01	3.73	4.03	51.92	0.00
Social Identity	Ioannis 2020	[30]	4.52	0.12	0.01	4.29	4.75	38.76	0.00
Social Identity	Martin and Horn 2013	[78]	6.38	0.06	0.00	6.26	6.50	101.18	0.00
Social Identity	Martin et al. 1995	[6]	4.25	0.26	0.07	3.74	4.76	16.45	0.00
Social Identity	Mitchell et al. 2014	[70]	5.47	0.06	0.00	5.36	5.58	97.12	0.00
Social Identity	Nagata and Long 2016	[51]	5.32	0.21	0.04	4.90	5.74	25.10	0.00
Social Identity	Peiró-Velert et al. 2016	[50]	4.83	0.08	0.01	4.66	5.00	57.31	0.00
Social Identity	Phoenix et al. 2005	[105]	5.32	0.13	0.02	5.06	5.58	39.62	0.00
Social Identity	Phoenix et al. 2005	[105]	5.32	0.14	0.02	5.04	5.60	37.81	0.00
Social Identity	Poczwardowski et al. 2014	[72]	6.11	0.27	0.07	5.58	6.64	22.68	0.00
Social Identity	Proios 2013	[76]	5.40	0.10	0.01	5.20	5.60	52.37	0.00
Social Identity	Rongen 2020	[31]	5.69	0.12	0.01	5.45	5.93	47.37	0.00
Social Identity	Samuel and Tenebaum 2011	[87]	5.98	0.06	0.00	5.87	6.09	104.61	0.00
Social Identity	Samuel et al. 2015	[57]	6.00	0.41	0.16	5.21	6.79	14.81	0.00
Social Identity	Tasiemski et al. 2013	[80]	5.72	0.23	0.05	5.26	6.18	24.60	0.00
Social Identity	Tasiemski et al. 2013	[80]	5.39	0.20	0.04	4.99	5.79	26.58	0.00
Social Identity	Weinberg et al. 2013	[75]	4.44	0.11	0.01	4.22	4.66	39.55	0.00

Supplement Files

Table S3. Individual study correlations.

[Ref#]	Study	AIMS Measure	N	r	SE	Fisher's Z	Correlate	Brewer Classification
[107]	Lau et al. 2004	Total	92	0.02	0.11	0.02	Amotivation, External Regulation	Not desired
[45]	O'Rourke et al. 2017	Total	70	−0.11	0.12	−0.11	Amotivation, External Regulation	Not desired
[45]	O'Rourke et al. 2017	Total	70	0.36	0.11	0.38	Amotivation, External Regulation	Not desired
[60]	Shutte and McNeil 2015	Total	400	0.4	0.04	0.42	Amotivation, External Regulation	Not desired
[90]	Gapin and Petruzello 2011	Exclusivity	179	−0.07	0.08	−0.07	Body Issues	Not desired
[90]	Gapin and Petruzello 2011	Exclusivity	179	0.1	0.07	0.1	Body Issues	Not desired
[90]	Gapin and Petruzello 2011	Exclusivity	179	0.12	0.07	0.12	Body Issues	Not desired
[113]	Martin 1999	Exclusivity	57	−0.16	0.13	−0.16	Body Issues	Not desired
[116]	Smith et al. 1998	Exclusivity	47	0.18	0.15	0.18	Body Issues	Not desired
[90]	Gapin and Petruzello 2011	Negative Affectivity	179	0.18	0.07	0.18	Body Issues	Not desired
[90]	Gapin and Petruzello 2011	Negative Affectivity	179	0.22	0.07	0.22	Body Issues	Not desired
[90]	Gapin and Petruzello 2011	Negative Affectivity	179	0.33	0.07	0.34	Body Issues	Not desired
[113]	Martin 1999	Negative Affectivity	57	−0.03	0.14	−0.03	Body Issues	Not desired
[90]	Gapin and Petruzello 2011	Self-Identity	179	0.06	0.08	0.06	Body Issues	Not desired
[90]	Gapin and Petruzello 2011	Self-Identity	179	0.01	0.08	0.01	Body Issues	Not desired
[90]	Gapin and Petruzello 2011	Self-Identity	179	−0.16	0.07	−0.16	Body Issues	Not desired
[113]	Martin 1999	Self-Identity	57	−0.47	0.11	−0.51	Body Issues	Not desired
[90]	Gapin and Petruzello 2011	Social Identity	179	−0.06	0.08	−0.06	Body Issues	Not desired
[90]	Gapin and Petruzello 2011	Social Identity	179	0.03	0.08	0.03	Body Issues	Not desired
[90]	Gapin and Petruzello 2011	Social Identity	179	0.06	0.08	0.06	Body Issues	Not desired
[113]	Martin 1999	Social Identity	57	−0.25	0.13	−0.26	Body Issues	Not desired
[116]	Smith et al. 1998	Social Identity	47	0.3	0.14	0.31	Body Issues	Not desired
[90]	Gapin and Petruzello 2011	Total	179	−0.03	0.08	−0.03	Body Issues	Not desired
[90]	Gapin and Petruzello 2011	Total	179	0.15	0.07	0.15	Body Issues	Not desired
[90]	Gapin and Petruzello 2011	Total	179	0.18	0.07	0.18	Body Issues	Not desired
[89]	Steinfeldt et al. 2011	Total	197	0.33	0.06	0.34	Body Issues	Not desired
[46]	Turton et al. 2017	Total	222	0.33	0.06	0.34	Body Issues	Not desired
[46]	Turton et al. 2017	Total	279	0.03	0.06	0.03	Body Issues	Not desired
[46]	Turton et al. 2017	Total	222	0.04	0.07	0.04	Body Issues	Not desired
[46]	Turton et al. 2017	Total	279	0.06	0.06	0.06	Body Issues	Not desired
[46]	Turton et al. 2017	Total	222	0.14	0.07	0.14	Body Issues	Not desired
[46]	Turton et al. 2017	Total	279	0.34	0.05	0.35	Body Issues	Not desired

Supplement Files

[68]	Voelker et al. 2014	Total	210	0.18	0.07	0.18	Body Issues	Not desired
[68]	Voelker et al. 2014	Total	210	0.01	0.07	0.01	Body Issues	Not desired
[68]	Voelker et al. 2014	Total	210	−0.03	0.07	−0.03	Body Issues	Not desired
[68]	Voelker et al. 2014	Total	210	0.26	0.06	0.27	Body Issues	Not desired
[68]	Voelker et al. 2014	Total	210	0.36	0.06	0.38	Body Issues	Not desired
[68]	Voelker et al. 2014	Total	210	0.14	0.07	0.14	Body Issues	Not desired
[68]	Voelker et al. 2014	Total	210	−0.22	0.07	−0.22	Body Issues	Not desired
[39]	Voelker et al. 2018	Total	29	0.07	0.2	0.07	Body Issues	Not desired
[39]	Voelker et al. 2018	Total	29	0.01	0.2	0.01	Body Issues	Not desired
[39]	Voelker et al. 2018	Total	29	0.15	0.19	0.15	Body Issues	Not desired
[101]	Kokaridas et al. 2009	Exclusivity	50	0.6	0.09	0.69	GO, Ego, Win	Not desired
[6]	Martin et al. 1995	Exclusivity	57	0.25	0.13	0.26	GO, Ego, Win	Not desired
[101]	Kokaridas et al. 2009	Negative Affectivity	50	0.4	0.12	0.42	GO, Ego, Win	Not desired
[6]	Martin et al. 1995	Negative Affectivity	57	0.15	0.13	0.15	GO, Ego, Win	Not desired
[6]	Martin et al. 1995	Self-Identity	57	0.27	0.13	0.28	GO, Ego, Win	Not desired
[101]	Kokaridas et al. 2009	Social Identity	50	0.27	0.14	0.28	GO, Ego, Win	Not desired
[6]	Martin et al. 1995	Social Identity	57	0.31	0.12	0.32	GO, Ego, Win	Not desired
[4]	Brewer et al. 1993	Total	449	0.34	0.04	0.35	GO, Ego, Win	Not desired
[52]	Haralabos et al. 2016	Total	150	0.16	0.08	0.16	GO, Ego, Win	Not desired
[107]	Lau et al. 2004	Total	92	0.35	0.09	0.37	GO, Ego, Win	Not desired
[45]	O'Rourke et al. 2017	Total	70	0.43	0.1	0.46	GO, Ego, Win	Not desired
[101]	Kokaridas et al. 2009	Exclusivity	50	0.39	0.12	0.41	GO, Mastery, Task	Desired
[6]	Martin et al. 1995	Exclusivity	57	0.19	0.13	0.19	GO, Mastery, Task	Desired
[101]	Kokaridas et al. 2009	Negative Affectivity	50	0.47	0.11	0.51	GO, Mastery, Task	Desired
[6]	Martin et al. 1995	Negative Affectivity	57	0	0.14	0	GO, Mastery, Task	Desired
[6]	Martin et al. 1995	Self-Identity	57	0.27	0.13	0.28	GO, Mastery, Task	Desired
[101]	Kokaridas et al. 2009	Social Identity	50	0.15	0.14	0.15	GO, Mastery, Task	Desired
[6]	Martin et al. 1995	Social Identity	57	0.23	0.13	0.23	GO, Mastery, Task	Desired
[4]	Brewer et al. 1993	Total	449	0.26	0.04	0.27	GO, Mastery, Task	Desired
[52]	Haralabos et al. 2016	Total	150	0.49	0.06	0.54	GO, Mastery, Task	Desired
[107]	Lau et al. 2004	Total	92	0.48	0.08	0.52	GO, Mastery, Task	Desired
[45]	O'Rourke et al. 2017	Total	70	0.35	0.11	0.37	GO, Mastery, Task	Desired
[24]	Hagiwara et al. 2020	Exclusivity	1514	0.74	0.01	0.95	Intrinsic Motivation, Commitment	Desired
[24]	Hagiwara et al. 2020	Negative Affectivity	1514	0.63	0.02	0.74	Intrinsic Motivation, Commitment	Desired

Supplement Files

[24]	Hagiwara et al. 2020	Social Identity	1514	0.76	0.01	1	Intrinsic Motivation, Commitment	Desired
[24]	Hagiwara et al. 2020	Total	1514	0.79	0.01	1.07	Intrinsic Motivation, Commitment	Desired
[112]	Horton and Mack 2000	Total	236	0.47	0.05	0.51	Intrinsic Motivation, Commitment	Desired
[107]	Lau et al. 2004	Total	92	0.39	0.09	0.41	Intrinsic Motivation, Commitment	Desired
[45]	O'Rourke et al. 2017	Total	70	0.38	0.1	0.4	Intrinsic Motivation, Commitment	Desired
[33]	Roethlisberger et al. 2020	Total	130	0.51	0.07	0.56	Intrinsic Motivation, Commitment	Desired
[60]	Shutte and McNeil 2015	Total	400	0.4	0.04	0.42	Intrinsic Motivation, Commitment	Desired
[107]	Lau et al. 2004	Total	92	0.42	0.09	0.45	Introjected, Identified Regulations	Desired
[60]	Shutte and McNeil 2015	Total	400	0.39	0.04	0.41	Introjected, Identified Regulations	Desired
[60]	Shutte and McNeil 2015	Total	400	0.26	0.05	0.27	Introjected, Identified Regulations	Desired
[78]	Martin and Horn 2013	Exclusivity	186	−0.24	0.07	−0.24	Negative Affect, Emotions, Feelings	Not desired
[78]	Martin and Horn 2013	Exclusivity	186	−0.02	0.07	−0.02	Negative Affect, Emotions, Feelings	Not desired
[78]	Martin and Horn 2013	Exclusivity	186	−0.23	0.07	−0.23	Negative Affect, Emotions, Feelings	Not desired
[29]	Graupensperger et al. 2020	Negative Affectivity	234	−0.05	0.07	−0.05	Negative Affect, Emotions, Feelings	Not desired
[78]	Martin and Horn 2013	Negative Affectivity	186	−0.22	0.07	−0.22	Negative Affect, Emotions, Feelings	Not desired
[78]	Martin and Horn 2013	Negative Affectivity	186	0.15	0.07	0.15	Negative Affect, Emotions, Feelings	Not desired
[78]	Martin and Horn 2013	Negative Affectivity	186	−0.1	0.07	−0.1	Negative Affect, Emotions, Feelings	Not desired
[78]	Martin and Horn 2013	Social Identity	186	−0.32	0.07	−0.33	Negative Affect, Emotions, Feelings	Not desired
[78]	Martin and Horn 2013	Social Identity	186	−0.02	0.07	−0.02	Negative Affect, Emotions, Feelings	Not desired
[78]	Martin and Horn 2013	Social Identity	186	−0.34	0.07	−0.35	Negative Affect, Emotions, Feelings	Not desired
[42]	Chang et al. 2018	Total	130	−0.4	0.07	−0.42	Negative Affect, Emotions, Feelings	Not desired
[47]	Geukes et al. 2017	Total	53	−0.07	0.14	−0.07	Negative Affect, Emotions, Feelings	Not desired
[44]	Giannone et al. 2017	Total	72	0.15	0.12	0.15	Negative Affect, Emotions, Feelings	Not desired
[44]	Giannone et al. 2017	Total	72	0.34	0.11	0.35	Negative Affect, Emotions, Feelings	Not desired
[44]	Giannone et al. 2017	Total	72	0.12	0.12	0.12	Negative Affect, Emotions, Feelings	Not desired
[99]	Maxwell and Visek 2009	Total	144	0.13	0.08	0.13	Negative Affect, Emotions, Feelings	Not desired
[45]	O'Rourke et al. 2017	Total	70	0.14	0.12	0.14	Negative Affect, Emotions, Feelings	Not desired
[45]	O'Rourke et al. 2017	Total	70	0.13	0.12	0.13	Negative Affect, Emotions, Feelings	Not desired
[45]	O'Rourke et al. 2017	Total	70	0.05	0.12	0.05	Negative Affect, Emotions, Feelings	Not desired
[45]	O'Rourke et al. 2017	Total	70	0.21	0.12	0.21	Negative Affect, Emotions, Feelings	Not desired
[73]	Petrie et al. 2014	Total	92	0.07	0.11	0.07	Negative Affect, Emotions, Feelings	Not desired
[73]	Petrie et al. 2014	Total	92	−0.01	0.11	−0.01	Negative Affect, Emotions, Feelings	Not desired
[60]	Shutte and McNeil 2015	Total	400	0.26	0.05	0.27	Negative Affect, Emotions, Feelings	Not desired
[46]	Turton et al. 2017	Total	279	0.46	0.05	0.5	Negative Affect, Emotions, Feelings	Not desired

Supplement Files

[46]	Turton et al. 2017	Total	222	0.43	0.06	0.46	Negative Affect, Emotions, Feelings	Not desired
[91]	Visek et al. 2010	Total	192	0.28	0.07	0.29	Negative Affect, Emotions, Feelings	Not desired
[91]	Visek et al. 2010	Total	358	0.26	0.05	0.27	Negative Affect, Emotions, Feelings	Not desired
[110]	Schmid and Seiler 2003	Exclusivity	939	0.19	0.03	0.19	Positive Affect, Emotions, Feelings	Desired
[29]	Graupensperger et al. 2020	Negative Affectivity	234	0.2	0.06	0.2	Positive Affect, Emotions, Feelings	Desired
[29]	Graupensperger et al. 2020	Negative Affectivity	234	0.18	0.06	0.18	Positive Affect, Emotions, Feelings	Desired
[110]	Schmid and Seiler 2003	Negative Affectivity	939	0.11	0.03	0.11	Positive Affect, Emotions, Feelings	Desired
[110]	Schmid and Seiler 2003	Social Identity	939	0.19	0.03	0.19	Positive Affect, Emotions, Feelings	Desired
[106]	Albion and Fogarty 2005	Total	226	−0.05	0.07	−0.05	Positive Affect, Emotions, Feelings	Desired
[112]	Horton and Mack 2000	Total	236	0.31	0.06	0.32	Positive Affect, Emotions, Feelings	Desired
[112]	Horton and Mack 2000	Total	236	0.39	0.06	0.41	Positive Affect, Emotions, Feelings	Desired
[6]	Martin et al. 1995	Total	62	−0.11	0.13	−0.11	Positive Affect, Emotions, Feelings	Desired
[6]	Martin et al. 1995	Total	62	−0.2	0.12	−0.2	Positive Affect, Emotions, Feelings	Desired
[6]	Martin et al. 1995	Total	62	−0.24	0.12	−0.24	Positive Affect, Emotions, Feelings	Desired
[6]	Martin et al. 1995	Total	62	−0.3	0.12	−0.31	Positive Affect, Emotions, Feelings	Desired
[103]	Mateos et al. 2008	Total	35	0.27	0.16	0.28	Positive Affect, Emotions, Feelings	Desired
[94]	Mateos et al. 2010	Total	35	0.27	0.16	0.28	Positive Affect, Emotions, Feelings	Desired
[33]	Roethlisberger et al. 2020	Total	130	0.38	0.08	0.4	Positive Affect, Emotions, Feelings	Desired
[110]	Schmid and Seiler 2003	Total	939	0.22	0.03	0.22	Positive Affect, Emotions, Feelings	Desired
[60]	Shutte and McNeil 2015	Total	400	0.19	0.05	0.19	Positive Affect, Emotions, Feelings	Desired
[60]	Shutte and McNeil 2015	Total	400	0.19	0.05	0.19	Positive Affect, Emotions, Feelings	Desired
[46]	Turton et al. 2017	Total	279	0.23	0.06	0.23	Positive Affect, Emotions, Feelings	Desired
[46]	Turton et al. 2017	Total	222	0.19	0.07	0.19	Positive Affect, Emotions, Feelings	Desired
[32]	Walsh et al. 2020	Total	158	0.36	0.07	0.37	Positive Affect, Emotions, Feelings	Desired
[48]	Fuller 2017	Exclusivity	168	−0.07	0.08	−0.07	Self Esteem, Competence, Worth	Desired
[113]	Martin 1999	Exclusivity	57	−0.22	0.13	−0.22	Self Esteem, Competence, Worth	Desired
[65]	Nagata 2014	Exclusivity	57	−0.43	0.11	−0.46	Self Esteem, Competence, Worth	Desired
[105]	Phoenix et al. 2005	Exclusivity	60	0.29	0.12	0.3	Self Esteem, Competence, Worth	Desired
[105]	Phoenix et al. 2005	Exclusivity	60	0.26	0.12	0.27	Self Esteem, Competence, Worth	Desired
[105]	Phoenix et al. 2005	Exclusivity	60	0.24	0.12	0.24	Self Esteem, Competence, Worth	Desired
[105]	Phoenix et al. 2005	Exclusivity	60	0.39	0.11	0.41	Self Esteem, Competence, Worth	Desired
[110]	Schmid and Seiler 2003	Exclusivity	939	0.03	0.03	0.03	Self Esteem, Competence, Worth	Desired
[48]	Fuller 2017	Negative Affectivity	168	−0.14	0.08	−0.14	Self Esteem, Competence, Worth	Desired
[113]	Martin 1999	Negative Affectivity	57	−0.2	0.13	−0.2	Self Esteem, Competence, Worth	Desired

Supplement Files

[105]	Phoenix et al. 2005	Negative Affectivity	60	0.11	0.13	0.11	Self Esteem, Competence, Worth	Desired
[105]	Phoenix et al. 2005	Negative Affectivity	60	0.09	0.13	0.09	Self Esteem, Competence, Worth	Desired
[105]	Phoenix et al. 2005	Negative Affectivity	60	0.22	0.13	0.22	Self Esteem, Competence, Worth	Desired
[105]	Phoenix et al. 2005	Negative Affectivity	60	0.22	0.13	0.22	Self Esteem, Competence, Worth	Desired
[110]	Schmid and Seiler 2003	Negative Affectivity	939	−0.04	0.03	−0.04	Self Esteem, Competence, Worth	Desired
[113]	Martin 1999	Self-Identity	57	0.47	0.11	0.51	Self Esteem, Competence, Worth	Desired
[48]	Fuller 2017	Social Identity	168	0.14	0.08	0.14	Self Esteem, Competence, Worth	Desired
[113]	Martin 1999	Social Identity	57	0.03	0.14	0.03	Self Esteem, Competence, Worth	Desired
[105]	Phoenix et al. 2005	Social Identity	60	0.34	0.12	0.35	Self Esteem, Competence, Worth	Desired
[105]	Phoenix et al. 2005	Social Identity	60	0.44	0.11	0.47	Self Esteem, Competence, Worth	Desired
[105]	Phoenix et al. 2005	Social Identity	60	0.42	0.11	0.45	Self Esteem, Competence, Worth	Desired
[105]	Phoenix et al. 2005	Social Identity	60	0.4	0.11	0.42	Self Esteem, Competence, Worth	Desired
[110]	Schmid and Seiler 2003	Social Identity	939	0.08	0.03	0.08	Self Esteem, Competence, Worth	Desired
[4]	Brewer et al. 1993	Total	90	0.06	0.11	0.06	Self Esteem, Competence, Worth	Desired
[4]	Brewer et al. 1993	Total	90	0.42	0.09	0.45	Self Esteem, Competence, Worth	Desired
[4]	Brewer et al. 1993	Total	90	0.15	0.1	0.15	Self Esteem, Competence, Worth	Desired
[4]	Brewer et al. 1993	Total	90	0.11	0.11	0.11	Self Esteem, Competence, Worth	Desired
[4]	Brewer et al. 1993	Total	449	−0.01	0.05	−0.01	Self Esteem, Competence, Worth	Desired
[4]	Brewer et al. 1993	Total	90	0.12	0.11	0.12	Self Esteem, Competence, Worth	Desired
[4]	Brewer et al. 1993	Total	90	−0.03	0.11	−0.03	Self Esteem, Competence, Worth	Desired
[4]	Brewer et al. 1993	Total	90	0.19	0.1	0.19	Self Esteem, Competence, Worth	Desired
[4]	Brewer et al. 1993	Total	90	−0.02	0.11	−0.02	Self Esteem, Competence, Worth	Desired
[4]	Brewer et al. 1993	Total	90	0.22	0.1	0.22	Self Esteem, Competence, Worth	Desired
[96]	Caudroit et al. 2010	Total	108	0.15	0.1	0.15	Self Esteem, Competence, Worth	Desired
[96]	Caudroit et al. 2010	Total	108	0.06	0.1	0.06	Self Esteem, Competence, Worth	Desired
[119]	Cornelius 1995	Total	224	0.04	0.07	0.04	Self Esteem, Competence, Worth	Desired
[59]	Houle and Kluck 2015	Total	250	0.12	0.06	0.12	Self Esteem, Competence, Worth	Desired
[55]	Huang et al. 2016	Total	345	0.01	0.05	0.01	Self Esteem, Competence, Worth	Desired
[111]	Kornspan and Etzel 2001	Total	259	−0.1	0.06	−0.1	Self Esteem, Competence, Worth	Desired
[107]	Lau et al. 2004	Total	92	0.79	0.04	1.07	Self Esteem, Competence, Worth	Desired
[58]	Poux and Fry 2015	Total	100	0.09	0.1	0.09	Self Esteem, Competence, Worth	Desired
[110]	Schmid and Seiler 2003	Total	939	0.03	0.03	0.03	Self Esteem, Competence, Worth	Desired
[91]	Visek et al. 2010	Total	358	0.26	0.05	0.27	Self Esteem, Competence, Worth	Desired
[91]	Visek et al. 2010	Total	192	0.28	0.07	0.29	Self Esteem, Competence, Worth	Desired

Supplement Files

[68]	Voelker et al. 2014	Total	210	−0.19	0.07	−0.19	Self Esteem, Competence, Worth	Desired
[39]	Voelker et al. 2018	Total	29	−0.07	0.2	−0.07	Self Esteem, Competence, Worth	Desired
